# Environment and bladder cancer: molecular analysis by interaction networks

**DOI:** 10.18632/oncotarget.18222

**Published:** 2017-05-26

**Authors:** Andrea Polo, Anna Crispo, Pellegrino Cerino, Luca Falzone, Saverio Candido, Aldo Giudice, Giuseppina De Petro, Gennaro Ciliberto, Maurizio Montella, Alfredo Budillon, Susan Costantini

**Affiliations:** ^1^ Epidemiology Unit, Istituto Nazionale Tumori “Fondazione G. Pascale”, IRCCS, Napoli, Italia; ^2^ Istituto Zooprofilattico Sperimentale del Mezzogiorno (IZSM), Napoli, Italia; ^3^ Department of Biomedical and Biotechnological Sciences, Section of General and Clinical Pathology and Oncology – Translational Oncology and Functional Genomics Laboratory, University of Catania, Catania, Italy; ^4^ Dipartimento di Medicina Molecolare e Traslazionale, Università di Brescia, Brescia, Italia; ^5^ Scientific Directorate, Istituto Nazionale Tumori “Regina Elena”, IRCCS, Roma, Italia; ^6^ Experimental Pharmacology Unit, Istituto Nazionale Tumori “Fondazione G. Pascale”, IRCCS, Napoli, Italia

**Keywords:** bladder cancer, network analysis, environmental exposure, arsenicals

## Abstract

Bladder cancer (BC) is the 9th most common cancer worldwide, and the 6th most common cancer in men. Its development is linked to chronic inflammation, genetic susceptibility, smoking, occupational exposures and environmental pollutants. Aim of this work was to identify a sub-network of genes/proteins modulated by environmental or arsenic exposure in BC by computational network approaches. Our studies evidenced the presence of HUB nodes both in “BC and environment” and “BC and arsenicals” networks. These HUB nodes resulted to be correlated to circadian genes and targeted by some miRNAs already reported as involved in BC, thus suggesting how they play an important role in BC development due to environmental or arsenic exposure. Through data-mining analysis related to putative effect of the identified HUB nodes on survival we identified genes/proteins and their mutations on which it will be useful to focus further experimental studies related to the evaluation of their expression in biological matrices and to their utility as biomarkers of BC development.

## INTRODUCTION

Bladder cancer (BC) is the 9th most common cancer worldwide, and the 4th most common cancer in more developed world [1–3]. In the last years, BC risk has been linked to the smoking, to the occupational exposures [4], to the use of drugs like cyclophosphamide or chlornaphazine [5], to the environmental pollutants and the arsenicals [6, 7], and to the chronic inflammation with the related up-regulation of some pro-inflammatory proteins like interleukin (IL)-6, tumor necrosis factor (TNF) and C-reactive protein (CRP) [8]. Moreover, a strong correlation between altered expression of various clock genes (PER1 and PER2) and common tumor markers (TP53, PTEN and PAI-1) evidenced that a disturbed function in the cellular clock system can clearly represent an another mechanism of BC progression [9].

In particular, many studies reported experimental evidences about the role of the environmental chemicals in BC carcinogenesis. In fact, the increased COX-2 expression and the activation of the mitogen activated protein kinase pathway in BC cells exposed to monomethylarsonous acid, a metabolite of inorganic arsenic, resulted a strong mechanism for BC carcinogenesis [10]. Moreover, it has been demonstrated in mice and rats the BC carcinogenicity of both 3-amino-1-methyl-5H-pyrido[4,3-b]indole, that is formed in cooked meat and fish [11], and pellets containing crude tryptophan pyrolysate and its derivates [12]. On the other hand, the exposure to high concentrations of diesel engine emissions, belonging to the sub-category of pollutants named as vehicle emissions, as well as insecticides, fungicides and pesticides resulted to contribute to carcinogenesis [13–15].

Our research group works in Campania, a region of Italy, in which BC incidence is very high with 75.3 cases per 100000 inhabitants, in comparison to only 19 cases per 100000 inhabitants in Europe in 2012 [3]. In the last decades, large areas of the Naples and Caserta provinces have been extensively contaminated by the widespread burial and open air dumping and incineration of industrial toxic waste [16]. Recently, the serum levels of arsenic measured in living subjects of five municipalities in the Naples province resulted to be higher than expected compared to national average [16].

Since the etiology of BC development is multi-factorial, only its early detection can reduce mortality. Hence, the development of new non-invasive biomarkers would benefit patients. Some reviews in literature report a summary of studies conducted through different omics approaches aimed to evaluate the transcriptome, the miRNome, the metabolome and the long non coding RNAs profiling involved in BC [16–21].

However, any detailed information is reported until now about what genes/proteins modulated by environmental exposure or by only arsenicals are involved in BC development or progression, how they are correlated, and what metabolic pathways are affected. Therefore, our aim was to select the genes/proteins modulated by environmental exposure (comprising arsenicals, pollutants, smoking, insecticides, fungicides and pesticides) or by only arsenicals in BC starting from Comparative Toxicogenomics Database (CTD), and to correlate them by a computational network approach. Through this method, we identified: i) the specific HUB nodes, that are nodes with a large degree and have connections with many other nodes, ii) the HUB-HUB interaction sub-network between HUB nodes that can be considered as specific of the correlation between BC and environment, and, hence, of the BC development due to environmental exposure, iii) the correlations between HUB nodes with circadian genes and miRNAs, iv) effect of HUB nodes on survival outcome of BC patients and their mutational status in this cancer. Therefore, further experimental studies could be focused on these HUB nodes to verify their utility as new diagnostic and/or prognostic biomarkers as well as potential targets for chemoprevention approach.

## RESULTS AND DISCUSSION

### Selection of environmental chemicals implicated in BC and the related modulated proteins

**Figure 1:**
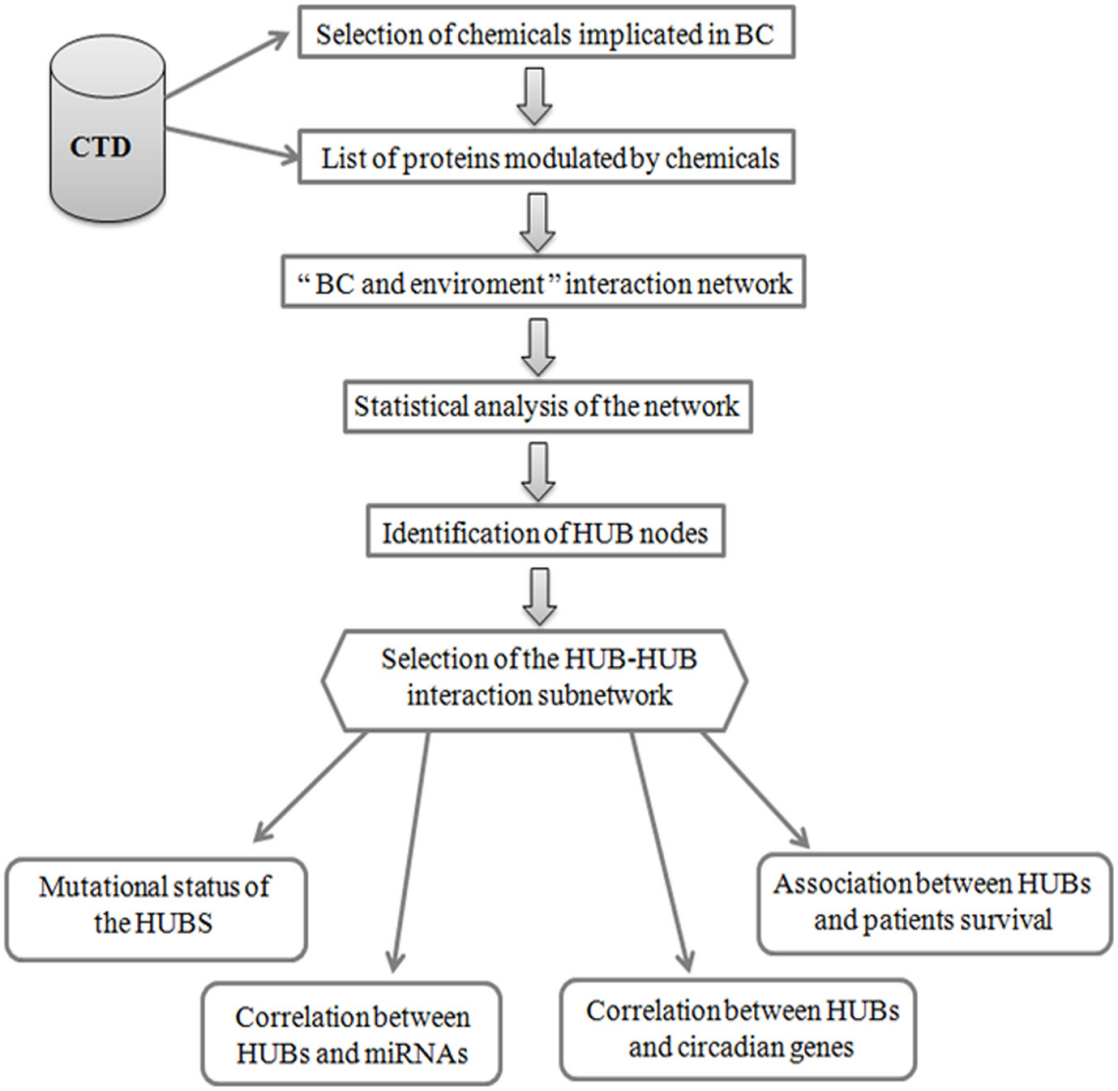
Flow-chart of the protocol used to identify and to analyse HUB nodes in “BC and environment” network

**Figure 2:**
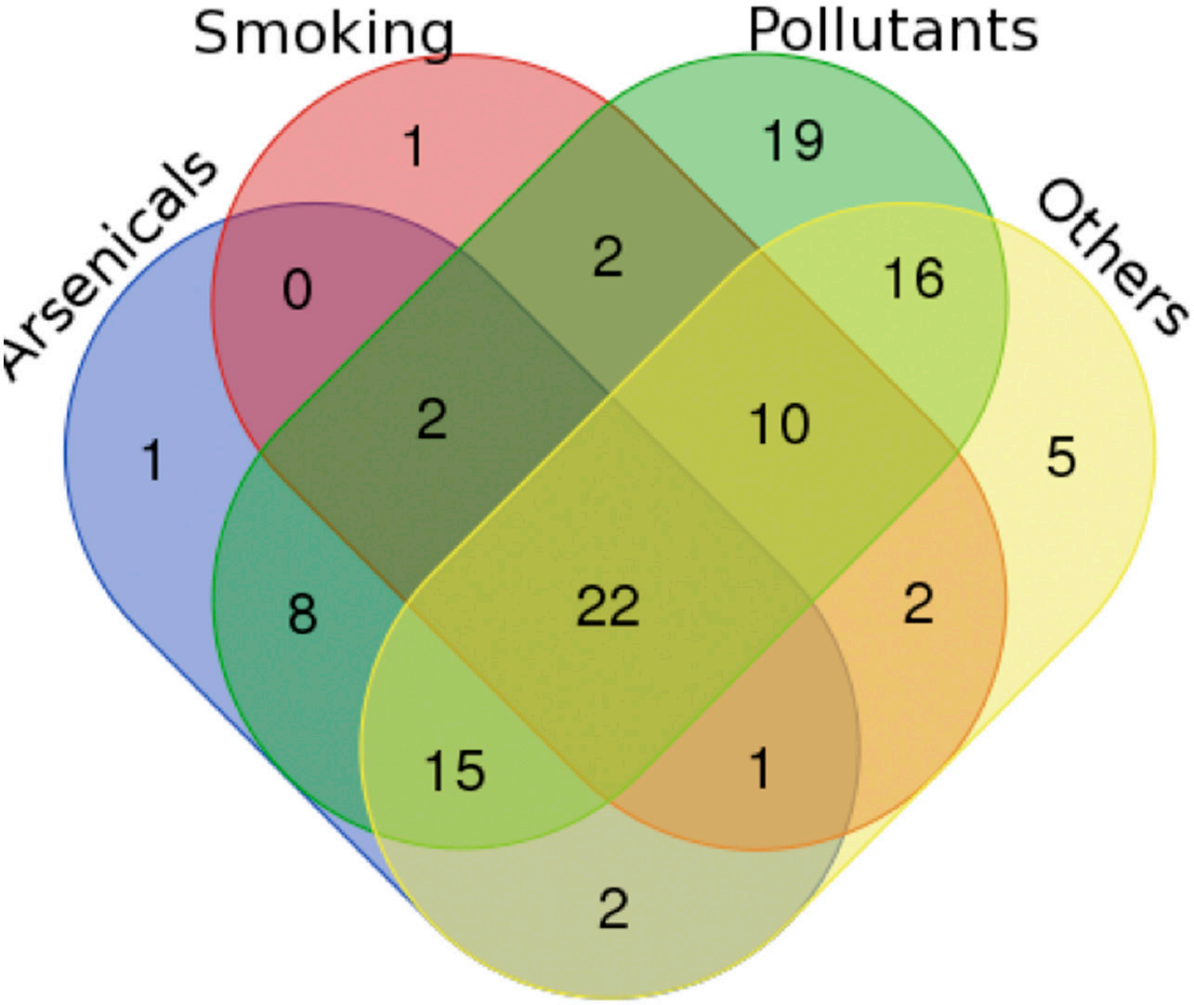
Venn diagram evidences the proteins that are modulated by four chemical sub-groups (arsenicals, smoking, pollutants and others) In this way it is possible to see the number of proteins modulated by only a sub-group or from two, three or four groups.

To create an interaction network between proteins modulated by environmental exposure in BC, we followed a protocol reported in Figure 1. Firstly we extracted the list of environmental chemicals implicated in BC and of the related proteins modulated by them using Comparative Toxicogenomics Database (CTD) [22]. The selected chemicals were grouped in the four following categories: arsenicals, smoking, pollutants and others, comprising insecticides, fungicides and pesticides, by considering also the studies already reported in literature [10–15] (Supplementary Table 1). About the identified proteins, we found 51 proteins modulated by arsenicals, 40 by smoking molecules, 94 by pollutants and 73 by other chemicals (Figure 2 and Supplementary Table 2). A molecular pathway analysis was conducted on all the proteins belonging to these four categories (Supplementary Table 3), and the molecular pathways, that were common or specific between the four categories, were extracted (Supplementary Table 4). In details, we can evidence the presence of some common pathways, that are strictly correlated to the cancer, among which: i) TNF and NOD-like receptor signaling pathway were common between arsenicals and smoking sub-groups; ii) Rap1, estrogen, prolactin, TGF-beta and VEGF signaling pathways were common between arsenicals, pollutants and “others” sub-groups; iii) hematopoietic cell lineage and Cytokine-cytokine receptor interaction molecular pathway were common between smoking and “others” sub-groups; iv) chemical carcinogenesis was common between smoking, pollutants and “others” sub-groups, and v) drug metabolism - cytochrome P450 was common between smoking and pollutants sub-groups. However, it is important to underline that, although different categories of chemicals are able to regulate similar pathways even if through different proteins along the same pathway, they can generate distinct molecular/cellular outputs.

Moreover, we evaluated also the pathways that are specific for each chemical category, and found for example: i) regulation of actin cytoskeleton, Wnt and Chemokine signaling pathway for arsenicals; ii) drug metabolism - other enzymes and metabolism of xenobiotics by cytochrome P450 for pollutants; iii) AK-STAT signaling pathway for “others” sub-group. Any specific molecular pathway for smoking was evidenced (Supplementary Table 4).

Moreover, a detailed analysis of the proteins modulated by only one chemical sub-category showed that FBXW8 was modulated only by arsenicals, ANXA2R only by smoking chemicals, and nineteen proteins (ASXL2, RALGPS1, STAG2, SMC1B, SMC1A, TYMP, SLC12A7, ARID1A, TACC3, APOBEC3B, MLL2, LOXL4, CALMHM1, NRSN1, EOMES, KMT2A, CHD6, VWA3A, KDM6A) only by pollutants (Figure 2 and Supplementary Table 2).

In details, FBXW8 is a member of the F-box protein family that acts in phosphorylation-dependent ubiquitination whereas ANXA2R may act as a receptor for annexin A2 that plays a role in the regulation of cellular growth and in signal transduction pathways. Regarding the nineteen proteins modulated by the pollutants, only STAG2, SMC1A and SMC1B resulted to be involved in the enriched molecular pathway named cell cycle.

However, through the Venn diagram analysis, we verified also the presence of twenty-two proteins that are modulated in BC by all four chemical sub-groups: CDH1, KRAS, IGFBP3, GSTM1, IGF1, TP53, IGFBP5, GSTP1, PTGS2, TNF, GPX1, CXCL8, CDKN2A, SOD2, EGFR, NQO1, MYC, CDKN1A, MT2A, BIRC3, FAS and ESR1 (Figure 2). These proteins are involved in the following molecular pathways: p53 signaling, MAPK signaling, Apoptosis, ErbB signaling, Cell cycle and Glutathione metabolism (Supplementary Table 5).

Since our principal aim was to identify a sub-network of genes/proteins modulated by environmental exposure in BC, we decided to focus our attention on the proteins modulated by all four chemical sub-groups and to create the general network of interaction between BC and environment.

### Identification and analysis of HUB nodes in “BC and environment” network

On the basis of the human molecular interactome and of the list of the twenty-two proteins, reported above, the “BC and environment” interaction network was created and analyzed. In detail, we mapped our twenty-two proteins on the human molecular interactome (INTACT) [23], extracted their related interaction network (named as “BC and environment”) composed by 1839 nodes and 2376 interactions and analyzed it by the related topological properties, reported in the Methods section, in order to understand the position and the role of these proteins present as nodes in the network (Supplementary Figure 1) [24]. In this way, we selected HUB nodes, which are the nodes with the strongest coordination role by considering a consensus of four out of six measures of centrality and topology (see Methods section). The following fifteen HUB nodes were identified: KRAS, IGFBP3, GSTM1, TP53, GSTP1, TNF, CXCL8, CDKN2A, SOD2, EGFR, NQO1, MYC, CDKN1A, FAS, and ESR1. They resulted to be up-regulated in BC by microarray studies with the exception of GSTP1 and ESR1 [25–31], and be involved in specific molecular functions and pathways (Supplementary Tables 5 and 6). We can speculate that the most part of identified HUB nodes is involved in molecular pathways correlated to cancer development and do not seem specific for BC. However, among these nodes, it is important to evidence the presence of GSTP1 and ESR1, about which few information is reported in BC. In general, GSTP1 catalyzes the conjugation of many hydrophobic and electrophilic compounds with reduced glutathione. It is reported that its polymorphisms are among the genetic determinants related to lead-induced inflammatory response and may modulate the response to epithelial oxidative changes caused by air pollutant exposure in lung [32]. Hence, it could be interesting and useful to study how GSTP1 is involved in BC development after environmental chemical exposure. In regard to ESR1, it is an estrogen receptor involved in sexual development and reproductive function. Considering that some environmental chemicals such as endosulfan and dieldrin are endocrine disruptors that can cause negative effects on the endocrine functions by miming the action of steroid hormones due to their structure similar to these last ones, we can think in future to investigate in more detail how ESR1 can be affected by these chemicals and through what mechanisms it can be involved in BC carcinogenesis.

About the obtained network a detailed analysis of the statistical centrality and topological measures has permitted to evidence its effectiveness and its robustness. In fact, it is important to underline that our network has a centralization value of 0.423, a network density of 0.001, a heterogeneity value of 9.899 and characteristic path length of 3.330. Overall these data evidenced that: i) the network effectiveness is elevated with nodes that are highly correlated between them; ii) the network is of small world type characterized by short path lengths [33]. Moreover, the plot of the node degree distribution showed a decreasing trend demonstrating that our network had scale free property indicating that it follows the role that “riches get richer” (Supplementary Figure 2) [34–37]. On the other hand the clustering coefficient graph showed a decreasing trend highlighting the tendency of our network to contain HUB nodes [37]. However, considering that the betweenness centrality is a measure to obtain inferences on the importance of inter-connected proteins on the basis of load placed on the given node in the network, the increasing trend of the betweenness centrality in our network demonstrated that the following five HUB nodes had the maximum load: i) TP53 (tumor protein p53), a DNA binding tumor suppressor protein, ii) MYC (c-MYC), a multifunctional and nuclear phosphoprotein that plays a role in cell cycle progression, apoptosis and cellular transformation, iii) EGFR (Epidermal Growth Factor Receptor), a cell surface receptor, iv) ESR1 (estrogen receptor), a nuclear hormone receptor and v) CDKN1A (Cyclin Dependent Kinase Inhibitor 1A) which functions as a regulator of cell cycle progression at G1.

Then, to study if in the network there were clusters and/or modules characterized by groups of nodes correlated between them, a cluster analysis was performed considering as statistically significant only the clusters with p-values lower than 0.001. In this way four clusters with density values ranging from 0.004 to 0.170 were selected (Supplementary Figure 7). They comprised639, 345, 68 and 25 proteins/nodes, respectively, and five HUB nodes. In detail, EGFR was in cluster 1, MYC in cluster 2, ESR1 in cluster 3, and GSTP1 and NQO1 in cluster 4. These clusters were named as EGFR cluster, MYC cluster, ESR1 cluster and GSTP1-NQO1 cluster, respectively. A functional analysis performed on the proteins present in the four clusters evidenced that each cluster comprised proteins involved in different metabolic pathways in comparison to those present in the other clusters. In fact, the only common pathways between at least two clusters were: i) “cell cycle and Epstein-Barr virus infection“ in the case of EGFR and MYC clusters, ii) “RNA transport” in the case of ESR1 and MYC clusters, and iii) “proteoglycans in cancer” in the case of EGFR and ESR1 clusters (Supplementary Table 7 and Supplementary Figure 4).

Overall the cluster analysis demonstrated that our “BC and environment” network comprised specific functional sub-networks in which some HUB nodes play crucial roles.

**Figure 3:**
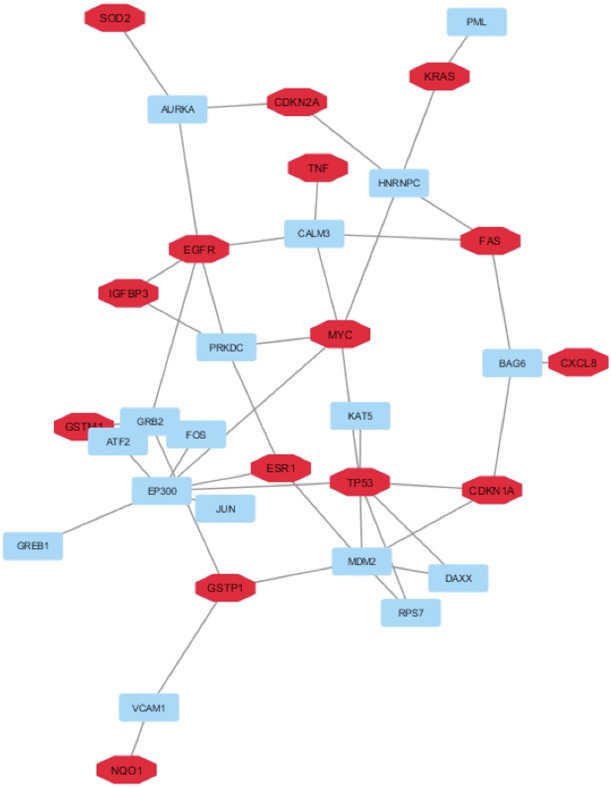
HUB – HUB interaction sub-network in “BC and environment” network In details, HUB nodes are reported in red whereas the other nodes in cyan.

Then, to select the sub-network of HUB nodes that were more correlated between them and to define the related HUB – HUB interaction sub-network, we extracted the interactions between HUB nodes in “BC and environment” network. In this way, it was possible to evidence that: i) four HUB nodes exhibited direct HUB–HUB interactions in the network (EGFR-IGFBP3 and TP53-CDKN1A) and ii) the other HUB nodes were linked between them through only one node (Figure 3). This finding suggested the strict functional relationship between these HUB nodes and how the HUB – HUB sub-network could represent a panel of proteins on which we can focus further studies in order to verify the possibility to use them as specific of the involvement of the environmental chemicals in BC initiation.

Moreover, the mutational status of the identified HUB nodes was evaluated. This analysis showed that: i) eight HUB nodes (ESR1, FAS, GSTM1, IFGBP3, KRAS, SOD2 and TNF) did not show mutations in BC, ii) CXCL8, NQO1 and MYC had one missense mutation, iii) GSTP1 had two missense mutations, iv) CDKN1A and EGFR had three missense mutations, v) CDKN2A had four missense mutations and vi) TP53 had thirty-eight missense mutations (Supplementary Table 8). Moreover, the co-occurrence of three mutations, i.e. T384S in EGFR and P151H in TP53, D136H in CDKN1A and R273S in TP53, and G146V in GSTP1 and G245S in TP53, resulted as statistically significant (p-value <0.001) suggesting their importance in BC initiation.

Then, to highlight in greater detail the involvement of these HUB nodes in BC development, we searched if they correlated with clock genes already reported to be altered in BC [9], and if they can be target of miRNAs reported to be implicated in BC [38].

In general, the circadian clock system comprises both negative and positive regulators, based on an auto-regulatory transcriptional and translational feedback program. In this context, PER and CRY proteins bind to the promoter region of BMAL1 and CLOCK, that are two transcription factors, and are capable to reduce the transcription of many genes during ambient light exposure [9]. In particular, the basic helix-loop-helix (bHLH)/PAS domain transcription factor plays a crucial role in the controlling the biological clock that controls the circadian rhythms.

Also the urinary system is regulated from the circadian rhythms. In fact, during day and night both urine excretion and extrusion are actively regulated by several internal factors and hormones. Such circadian variations led us to postulate that similar to other organs, the perturbation of the clockwork may contribute to the dysregulation that develops during BC development [9]. Since clock genes are able to modify the gene regulation, they may interact with the transcription of oncogenes and/or tumour suppressor-genes. In fact, a recent paper reported the close correlation between altered expression of various clock genes and common tumor markers in BC evidencing how a disturbed function in the cellular clock may be an important additional mechanism contributing to cancer progression [9]. Hence, we searched if there were correlations between the proteins codified from circadian genes (PER1, PER2, PER3, CRY1, CRY2, BMAL1, CLOCK, ANTI-CSNK1α1L, CSNK1α and CSNK1ε) and our HUB nodes. As visible in Supplementary Figure 5, there was a direct correlation between TP53 (HUB node) and CSNK1ε (circadian node) and other circadian nodes correlated with the other HUB nodes through one or two nodes. This demonstrated the strict relationship between circadian rhythms and HUB nodes and confirmed how our HUB nodes can have an important role in BC development in according to the other previous analysis.

**Figure 4:**
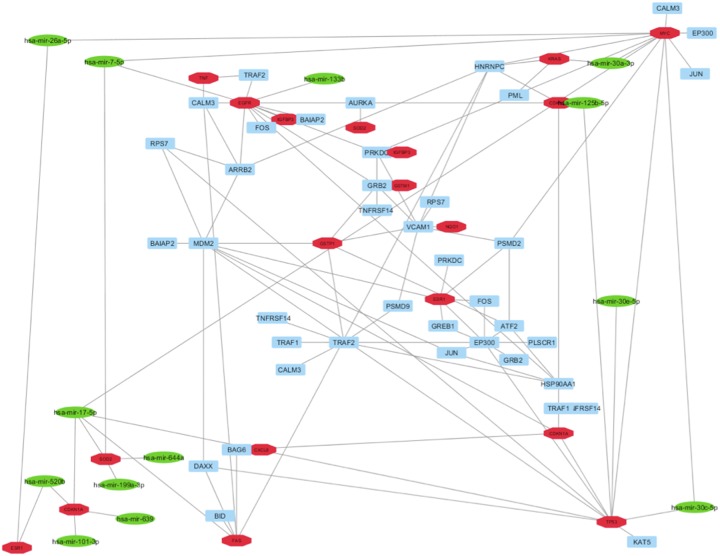
Correlation between HUB – HUB interaction sub-network in “BC and environment” network and miRNAs reported as implicated in BC in literature In details, HUB nodes are reported in red, miRNAs in green and other nodes in cyan.

The list of the miRNAs implicated in BC by microarray studies and verified experimentally by RT-PCR, and the list of the genes targeted from these miRNAs was extracted by mirNET database (Supplementary Table 9) [38]. In this way, it was possible to select only the miRNAs able to target our HUB nodes. In detail, our analysis evidenced that thirteen miRNAs (hsa-mir-7-5p, hsa-mir-17-5p, hsa-mir-26a-5p, hsa-mir-30a-3p, hsa-mir-30c-5p, hsa-mir-30e-5p, hsa-mir-101-3p, hsa-mir-125b-5p, hsa-mir-133b, hsa-mir-199a-3p, hsa-mir-520b, hsa-mir-639 and hsa-mir-644a) correlated with seven HUB nodes (EGFR, SOD2, MYC, KRAS, ESR1, CDKN1A and TP53) (Figure 4).

In addition, starting from these data, to understand if our HUB nodes can have a clinical validity and utility, we decided to perform a set of bioinformatics analyses on available gene expression datasets BC (TCGA-BLCA). The purpose was to assess if there was an association between the expression in BC of our HUB nodes and patients survival. Our analysis evidenced that high expression of EGFR, TP53, MYC, GSTP1, NQO1 and KRAS as well as the association of high co-expression of TP53-EGFR, MYC-EGFR, KRAS-EGFR, NQO1-EGFR, TP53-MYC had a negative effect on the survival (Table 1 and Supplementary Figure 6).

**Table 1:** Overall survival related to high expression/high co-expression of HUB nodes in the “BC and environment” and “BC and arsenicals” networks by SynTarget online tool using public TCGA_BLCA dataset (Bladder Urothelial Carcinoma)^31^

Nodes	Suvival effect	p-Value
**BC and environment network**		
EGFR	Negative	0.00124
TP53	Negative	0.00898
MYC	Negative	0.0276
GSTP1	Negative	0.0478
NQO1	Negative	0.0379
KRAS	Negative	0.017
TP53-EGFR	Negative	0.000301
MYC-EGFR	Negative	5.23e-06
KRAS-EGFR	Negative	0.000591
NQO1-EGFR	Negative	0.000134
TP53-MYC	Negative	0.00835
**BC and arsenicals network**		
KRAS	Negative	0.017
ERCC4	Negative	0.00424
ERCC4-KRAS	Negative	0.0385

The p-values lower than 0.05 were considered as statistically significant.

### Identification and analysis of HUB nodes in “BC and arsenicals” network

Since BC incidence is very high in the Campania region in Italy [3] and the SEBIOREC study demonstrated an higher level of arsenic in the serum of subjects living in the province of Napoli compared to national average [16], a particular attention was directed to establish an interaction between arsenicals and fifty-one proteins known to be modulated by them (Figure 2). In this study we followed the same protocol used in the case of “BC and environment” network (Figure 1). As reported in the first Results paragraph, the functional analysis on these proteins evidenced that they were involved in a set of molecular pathways (Supplementary Table 3). These fifty-one proteins were mapped on the human molecular interactome and the related interaction network named “BC and arsenicals” network was extracted and analyzed by different topological properties, as reported above for the “BC and environment” interaction network (Supplementary Figure 7) [24].

This network comprised 353 nodes (proteins) and 378 interactions (edges), and the following nine HUB nodes: PSMB2, TNF, BIRC3, FANCA, KRAS, CCNE1, ERCC4, PABPC1, and PRSS3. It is important to evidence that two HUB nodes, TNF and KRAS, were in common with the “BC and environment” interaction network whereas the others are uniquely regulated by arsenicals. Notably, all the HUB nodes, at exception of BIRC3 and FANCA, were already resulted to be up-regulated in BC by microarray studies [25–31, 39–43], and be involved in specific molecular functions (Supplementary Table 10). However, among the proteins already studied in BC, PSMB2 is a very interesting node; in fact, it is hypermethylated in BC and was identified as indicator of adverse health effects associated with arsenic exposure [44]. On the other hand, very few information is reported about BIRC3 and FANCA in BC. In particular, BIRC3 inhibits apoptosis by binding to tumor necrosis factor receptor-associated factors, and is dysregulated in some cancers, and FANCA is a DNA repair protein that may operate in a post-replication repair or a cell cycle checkpoint function. Notably, it could be interesting to study in future how these two proteins can be affected by arsenicals and through what mechanisms they can be involved in BC carcinogenesis.

A detailed analysis of the obtained network showed that it had a good centralization equal to 0.402 and a network density value of 0.006. The characteristic path length of 3.752 confirmed that also this network followed the small-world rule [32] as we evidenced for “BC and environment” network. Moreover, we can underline also that: i) the decreasing trend of the node degree distribution plot indicated that our network had scale free property with the occurrences of modules (Supplementary Figure 8A) [26–29], ii) the decreasing trend of the clustering coefficient graph showed the tendency of our network to contain HUB nodes (Supplementary Figure 8B) [43], and iii) the increasing trend of the betweenness centrality demonstrated the presence of a HUB node with maximum load like PABPC1 (Polyadenylate-Binding Protein 1) that is involved in cytoplasmic regulatory processes of mRNA metabolism such as pre-mRNA (Supplementary Figure 8C).

Then, to study if in the “BC and arsenicals” network there were modules characterized by groups of nodes correlated between them, a cluster analysis was performed as in the case of “BC and environment” network. This showed the presence of two clusters (with significant P-values lower than 0.005) comprising 15 and 10 proteins/nodes with density values ranging from 0.608 to 0.644 and three HUB nodes (Supplementary Figure 9). In detail, CCNE1 was in cluster 1 whereas TNF and BIRC3 were in cluster 2. However, cluster 1 comprised also CDKN1A, a HUB node related to “BC and environmental” network. The proteins present in cluster 1 resulted to be involved in specific metabolic pathways such as PI3K-Akt signaling pathway, p53 signaling pathway and cell cycle, whereas those in cluster 2 in Apoptosis, NF-kappa B signaling pathway, TNF signaling pathway and Adipocytokine signaling pathway. However, the only common pathway between two clusters is “Pathways in cancer“ (Supplementary Table 11 and Supplementary Figure 10).

As in the case of “BC and environment” network, cluster analysis highlighted that the “BC and arsenicals” network comprised two sub-networks in which the identified HUB nodes played important functional roles.

**Figure 5:**
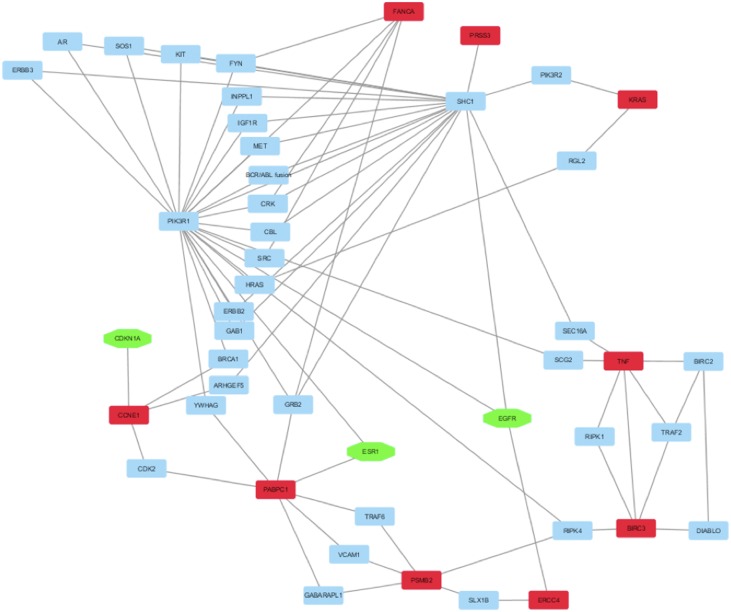
HUB – HUB interaction sub-network in “BC and arsenicals” network In details, HUB nodes are reported in red, HUB nodes that were present also in “BC and environmental network” in green and the other nodes in cyan.

Moreover, to select the HUB – HUB interaction sub-network in “BC and arsenicals” network, we focused on the interactions between HUB nodes, and evidenced that two HUB nodes (TNF-BIRC3) exhibited direct HUB–HUB interactions in network whereas the other HUB nodes were linked among them through one or two nodes (Figure 5). Moreover, three HUB nodes identified for the arsenicals (ERCC4, PABPC1 and CCNE1) resulted to interact directly with three HUB nodes evidenced in the “BC and environment” network (ERCC4-EGFR, PABPC1-ESR1, and CCNE1-CDKN1A) suggesting the strict correlation between “BC and arsenicals” and “BC and environment” networks.

However the analysis of HUB-HUB interaction sub-network in “BC and arsenicals” network evidenced how, with the exception of TNF and KRAS being in common between two networks, the remaining seven HUB nodes (PSMB2, BIRC3, FANCA, CCNE1, ERCC4, PABPC1, and PRSS3) can be considered as specific of the arsenicals involvement in BC development.

The analysis of the mutational status of all the HUB nodes, showed that: i) six HUB nodes (CCNE1, KRAS, PABPC1, PRSS3, PSMB2 and TNF) had not mutations in BC, ii) BIRC3, ERCC4 and FANCA had one, two and four missense mutations, respectively (Supplementary Table 12). No co-occurrence mutations resulted statistically significant.

Also in the case of “BC and arsenicals network”, we evaluated if there was a correlation between our HUB nodes and the clock genes resulted to be altered in BC [9]. As visible in Supplementary Figure 11, there was no direct correlation between HUB nodes and circadian nodes but the circadian nodes correlated with the HUB nodes through one or two nodes. This suggested that the relationship between circadian rhythms and HUB nodes in “BC and arsenicals” network was weaker in comparison to what observed for the “BC and environment” network.

**Figure 6:**
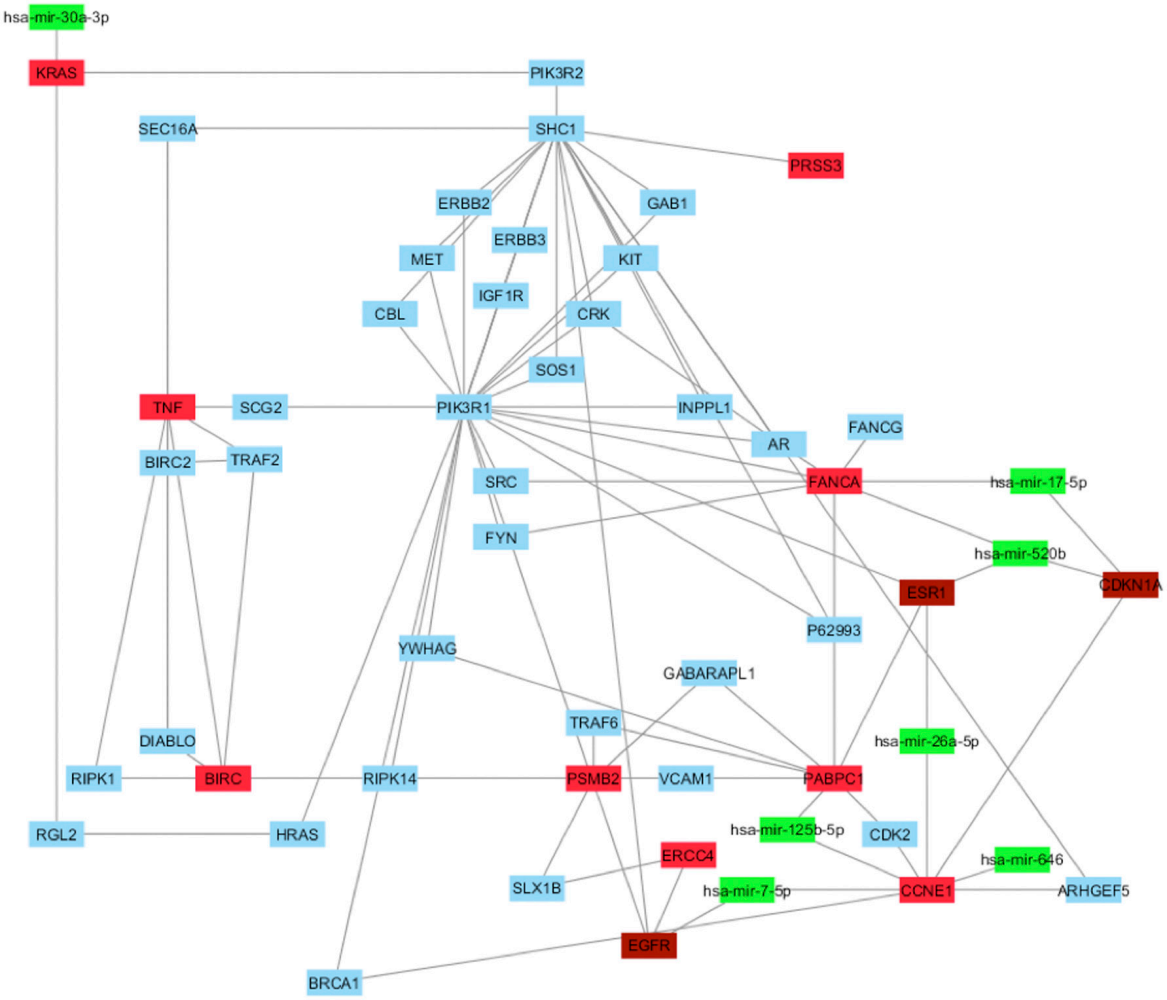
Correlation between HUB – HUB interaction network in “BC and arsenicals” network and miRNAs In details, HUB nodes are reported in red, miRNAs in green, HUB nodes present in “BC and environment” in brown and other nodes in cyan.

Then, we evaluated if our HUB nodes can be targets of miRNAs, resulted already as implicated in BC using the same protocol reported above. This analysis evidenced that seven miRNAs (hsa-mir-7-5p, hsa-mir-17-5p, hsa-mir-26a-5p, hsa-mir-30a-3p, hsa-mir-125b-5p, hsa-mir-520b and hsa-mir-646) correlated with four HUB nodes (FANCA, CCNE1, KRAS and PABPC1) (Figure 6 and Supplementary Table 13).

Among these miRNAs, only hsa-mir-646 did not correlate with the HUB nodes of “BC and environmental” network. Hence, it could be specific of the involvement of arsenicals in BC development.

Finally, we evaluated if there was an association between the expression of our HUB nodes in BC and patients survival as in the case of “BC and environmental” network. Our analysis showed that only the high expression of ERCC4 and KRAS and their high co-expression (ERCC4-KRAS) resulted to have a negative effect on the survival in BC patients (Supplementary Figure 12 and Table 1).

## CONCLUSIONS

No many papers were already published about the direct linking between the environmental or arsenic exposure and the related modulated proteins in BC. For example, it has been shown that arsenic exposure is positively associated with PRSS3 promoter methylation levels in BC [45] whereas the effect of NQO1 Pro187Ser polymorphism resulted to be more important in never smokers because no consistent results were obtained about tobacco-related BC risk [46]. Therefore, aim of this work was to highlight the correlation between genes and proteins that are modulated by environmental exposure or only by the arsenicals by network approaches, and to identify the related HUB nodes that can be considered as specific of the BC development due to environmental or arsenicals exposure, and on which can be useful to focus further experimental studies in order to verify their utility as new diagnostic and/or prognostic biomarkers or targets.

In summary, in our study we reported the creation of two networks named as “BC and environment” and “BC and arsenicals”, defined a set of HUB nodes and the related HUB-HUB interactions, and evidenced that these HUB nodes showed significant mutations implicated in BC and correlated with genes involved in circadian rhythms. In addition, we identified: i) a sub-network of interactions between miRNAs and genes that is specific of the correlation between BC and environment or arsenicals, and ii) the genes correlated to negative effect on the survival in BC patients.

On the basis of our results, we can underline that many identified HUB nodes in “BC and environment” and “BC and arsenicals” networks are proteins already known as to be involved in molecular pathways correlated to the development of cancer and BC. However, we evidenced also the presence of other proteins for which molecular interactions in BC were unknown. For example in “BC and environment” network we found the estrogen receptor, ESR1, that can be object of further investigations because some environmental chemicals are endocrine disruptors that can mimic its structure. Hence, this finding can cause problems in the endocrine system and could represent an initiation point for BC. Moreover, in the case of “BC and arsenicals” network, we identified two HUB nodes, BIRC3 and FANCA for which the role in BC is unknown. Therefore, it can be useful to study if and how these two proteins can be affected by arsenicals and if there are specific mechanisms through which they can contribute to BC carcinogenesis. Certainly these hypothesis may be verified by experimental studies but could represent starting points for the identification of new markers for BC.

Finally, it is important to underline that all our data fall within the SPES project (http://spes.campaniatrasparente.it/), in which our group is involved, that is an exposure study in susceptible population. Its aim is to evaluate the effects on human health of different sources of contamination. Through the analysis of the spatial distribution of the sources of contamination and the concentration values of exposure, genetic susceptibility, immune and oxidative biomarkers in biological fluids of youth living groups in the “Land of Fires” municipalities, it will be possible to identify areas that have the same potential index of risk. Therefore, the SPES project is collecting sera from healthy donors/patients of susceptible population to contamination, and, hence, the network studies in this work can provide knowledge useful for further hypothesis-driven experimental studies and targets discovery in BC. In fact, the identification of chemicals-regulated proteins could help to search for specific markers in selected populations. Therefore, considering the results of this study, we could think to evaluate: i) the expression of TP53 and EGFR, and their mutations (T384S of EGFR and P151H of TP53) in sera of susceptible individuals to the environmental chemicals exposure to verify if they can be used as markers for BC and ii) the expression of ERCC4, KRAS and of hsa-mir-646 in individuals exposed to contamination of drinking water with arsenicals to understand if and what among these three molecules can be used as markers for BC initiation.

## MATERIALS AND METHODS

### Network analysis

Comparative Toxicogenomics Database (CTD) was used to extract the list of environmental chemicals implicated in BC and the related chemical–protein interactions [22]. Through the Cytoscape software platform for the visualization of complex networks and their integration (http://www.cytoscape.org/), a network related to the interactions between the proteins modulated by the selected environmental chemicals was constructed using as reference the human molecular interactome (INTACT) [23] where all interactions are derived from literature curation or direct user submissions and are freely available. Some statistical analyses were performed the following three measures of centrality: i) the degree that indicates the number of interactions of a particular node with other nodes in the network; ii) the betweenness centrality that evaluates the importance of a node in the network and how the other interactions in the network are controlled by this node [47]; and iii) the closeness centrality of a node that is calculated as the sum of the length of the shortest paths between the node and all other nodes in the graph and ranges from 0 to 1 [48]. Then, we evaluated also other topological analyses like average characteristic path length, network density, and centralization [48–50]. The characteristic path length is calculated by finding the shortest path between all pairs of nodes, adding them up, and then dividing by the total number of pairs. This indicates the number of steps that takes to get from one member of the network to another. The density of a network is defined as a ratio of the number of edges to the number of possible edges [48] whereas the centralization produces rankings, which seek to identify the most important nodes in a network model ranging from 1 to 0 [49].

Finally, we performed a cluster analysis by means of Cluster-One [50] that is the task of grouping a set of objects in such a way that objects in the same group (called a cluster) are more similar to each other than to those in other groups (clusters) [51].

Functional and Pathway Analyses were performed by DAVID program [52].

### miRNA evaluations

The miRNAs able to target the HUB nodes were selected by MirNet tool [53]. In details, we performed the following protocol: firstly, we extracted the list of miRNAs involved in BC; secondly, starting from this miRNA list we extracted all their targets; finally we selected only the miRNAs that targeted our HUB nodes. Then, using the list of our HUB nodes and the related miRNAs, an interaction network between selected miRNAs and HUB nodes was constructed by the Cytoscape package.

### Survival gene effect analysis

Bionformatic analyses were performed by SynTarget online tool able to test the synergetic effect of genes on survival outcome in cancer (http://www.bioprofiling.de) using public TCGA_BLCA dataset (Bladder Urothelial Carcinoma) [53]. In details, PPISURV tool was used to test if our genes can be used as biomarker of cancer survival.

### Gene mutation analysis

Mutation analysis was conducted by cBioPortal for Cancer Genomics tool able to analyze cancer genomics data in order to test the presence of mutations on genes involved in cancer (http://cbioportal.org) [54, 55].

## SUPPLEMENTARY MATERIALS FIGURES AND TABLES




