# Supplementary material for: Environment and bladder cancer: molecular analysis by interaction networks
Source: Oncotarget. 2017 May 26;8(39):65240–52. doi: 10.18632/oncotarget.18222 (PMC5630327; doi:10.18632/oncotarget.18222)
Supplement: Supplementary file 1 [file oncotarget-08-65240-s001.pdf]

Environment and bladder cancer: molecular analysis by interaction networks

SUPPLEMENTARY MATERIALS

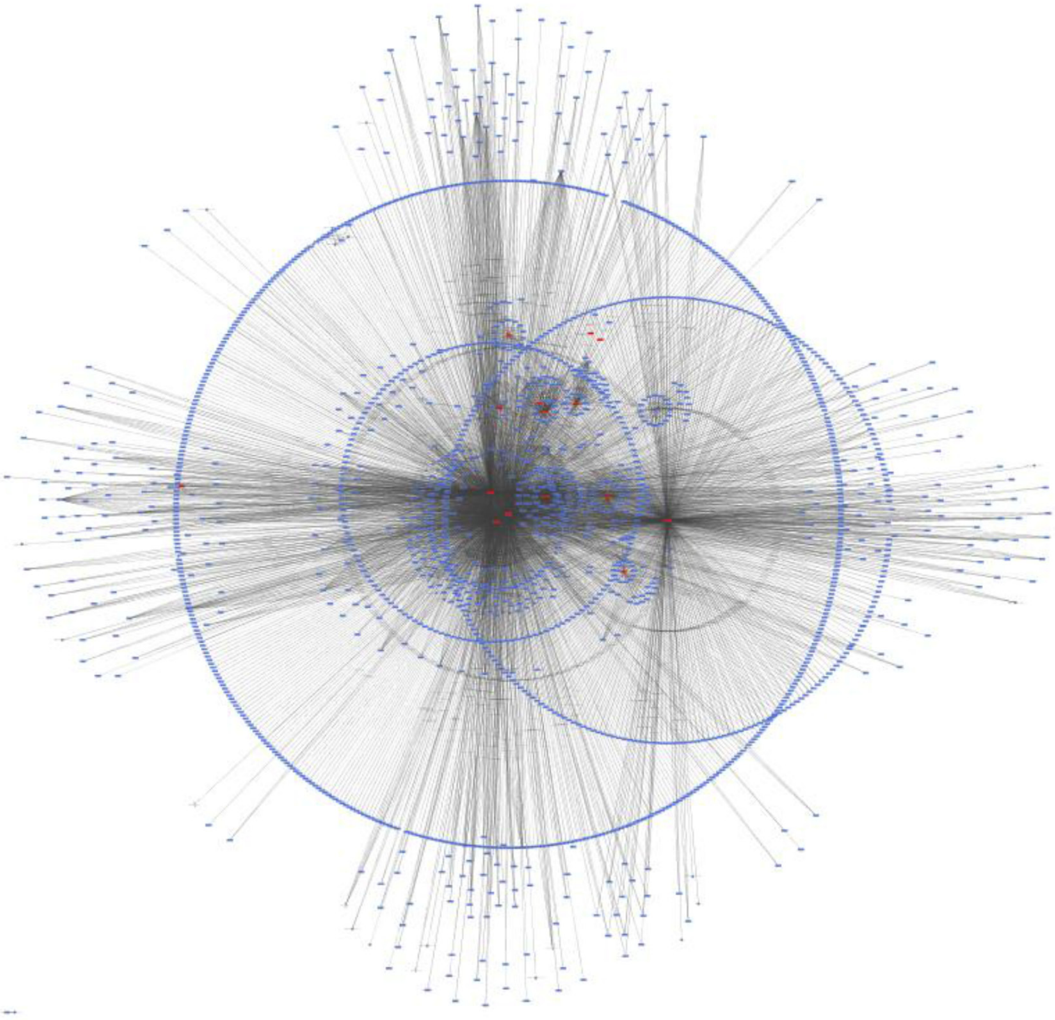

Supplementary Figure 1: Network related to the correlation between proteins modulated from environmental chemicals in BC. HUB nodes are reported in red.

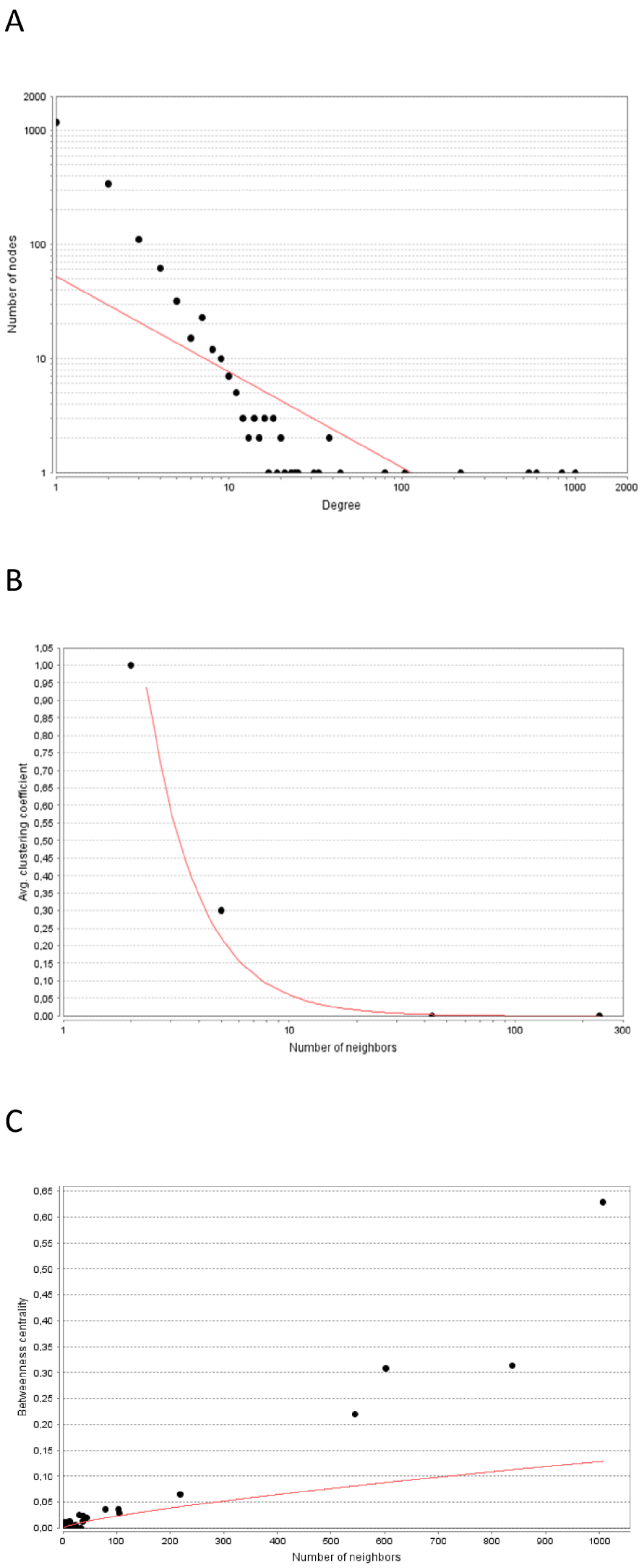

Supplementary Figure 2: Evaluation of topological properties of our network. **(A)** node degree distribution, **(B)** average clustering coefficient, and **(C)** betweenness centrality measure.

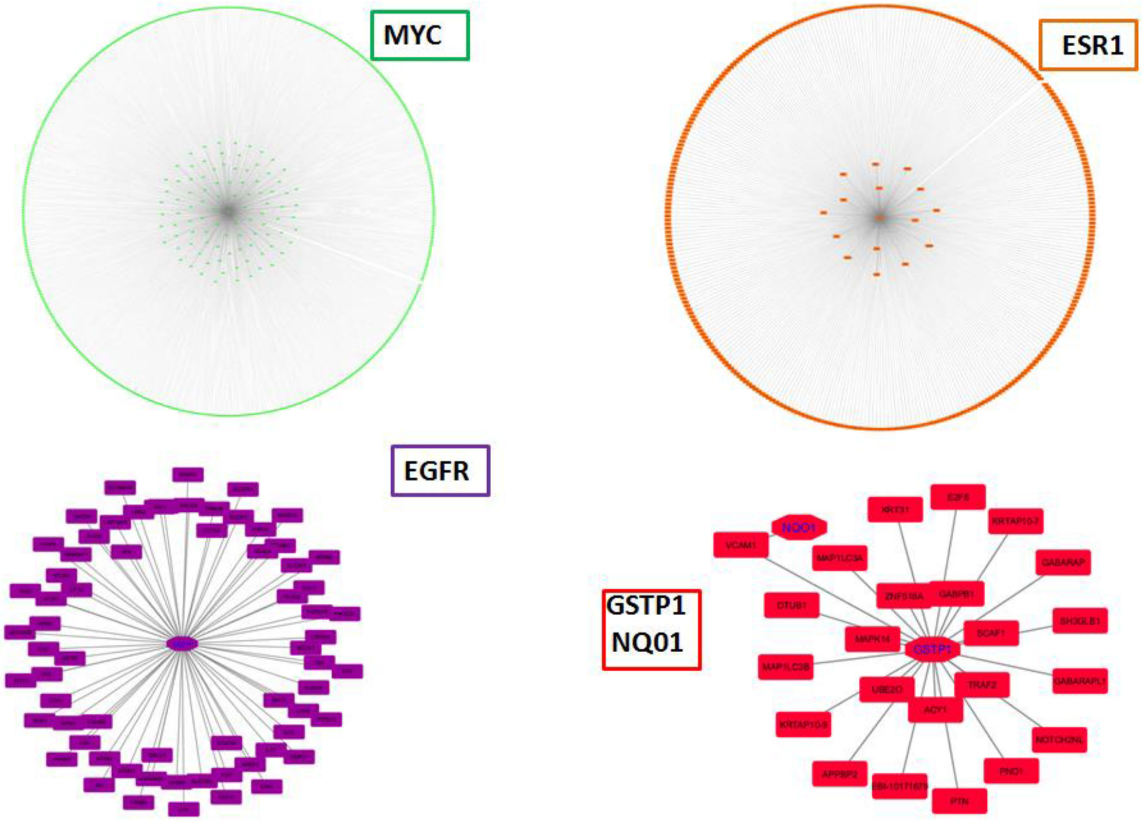

Supplementary Figure 3: Four clusters obtained starting the entire network reported in Supplementary Figure 1.

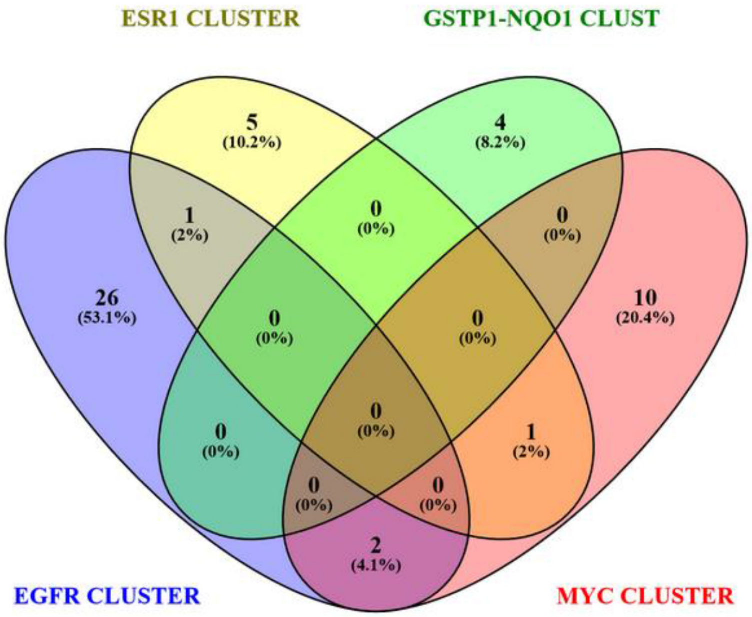

Supplementary Figure 4: Venn diagram evidences the number of common and specific pathways in which the proteins present in four clusters are involved.

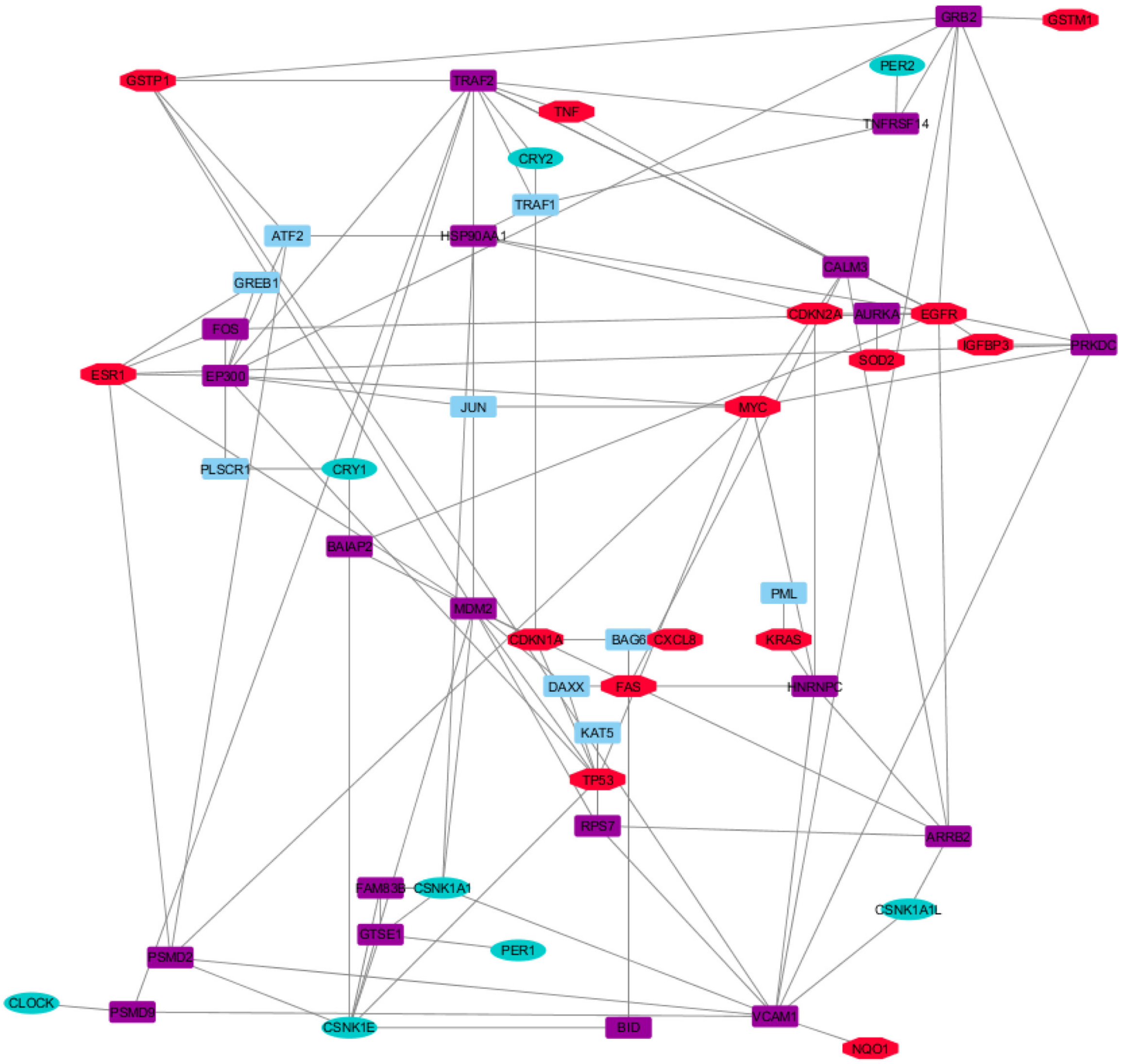

Supplementary Figure 5: Relationship between the HUB-HUB interaction network and circadian nodes. In details, we report HUB nodes in red, circadian nodes in blue marine, nodes linking HUB and circadian nodes in purple and the other nodes in blue.

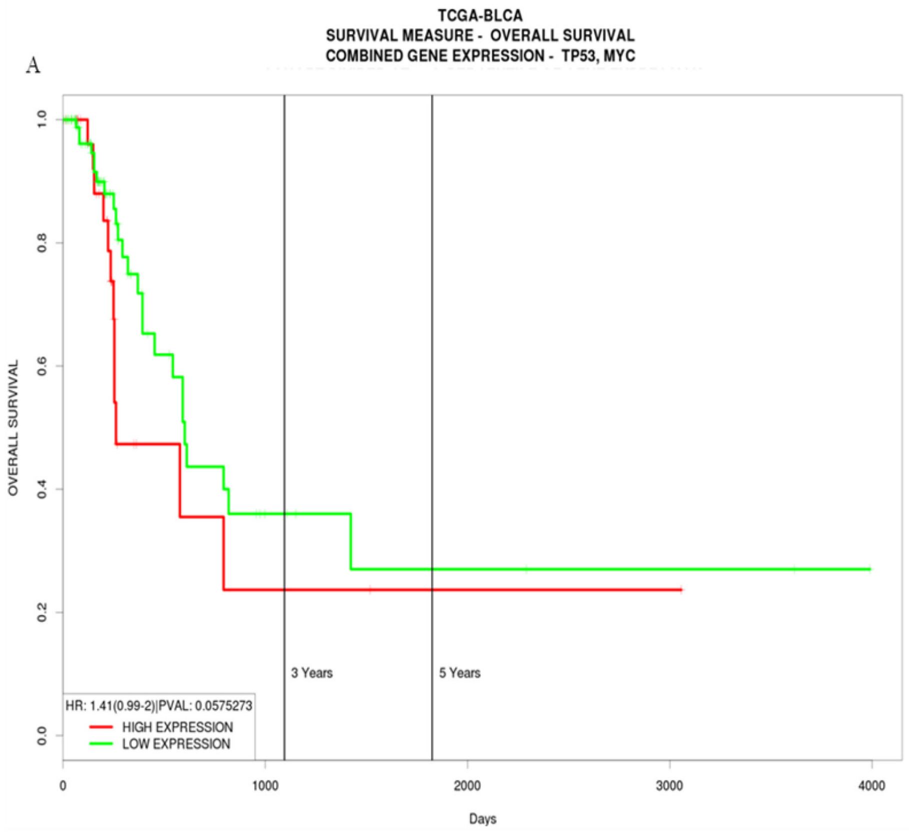
TCGA-BLCA
SURVIVAL MEASURE - OVERALL SURVIVAL
COMBINED GENE EXPRESSION - TP53, MYC
A
OVERALL SURVIVAL
Days
3 Years
5 Years
HR: 1.41(0.99-2)|PVAL: 0.0575273
HIGH EXPRESSION
LOW EXPRESSION

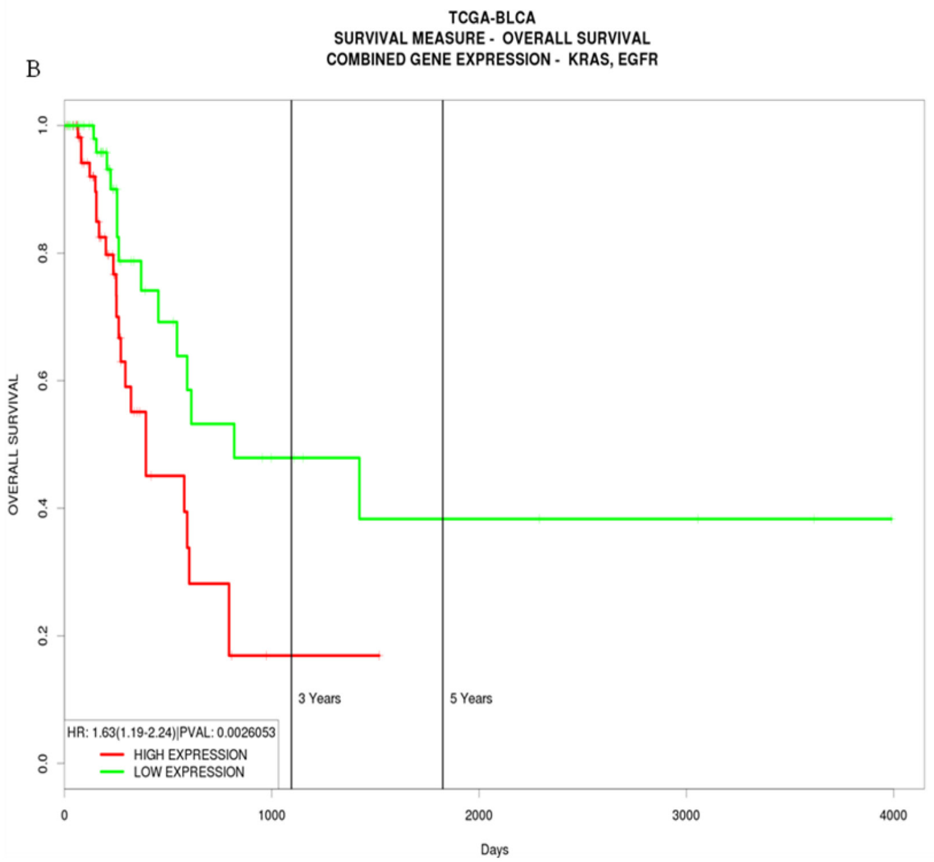
TCGA-BLCA
SURVIVAL MEASURE - OVERALL SURVIVAL
COMBINED GENE EXPRESSION - KRAS, EGFR
B
OVERALL SURVIVAL
Days
3 Years
5 Years
HR: 1.63(1.19-2.24)|PVAL: 0.0026053
HIGH EXPRESSION
LOW EXPRESSION

(*Continued*)

C

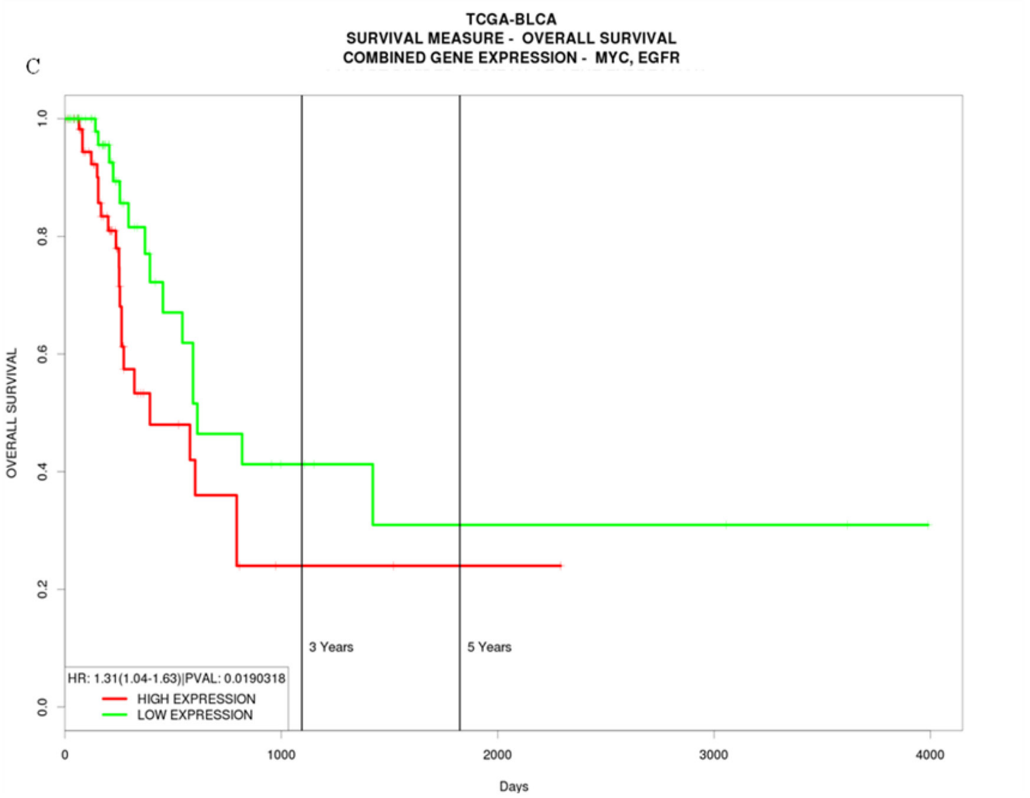

D

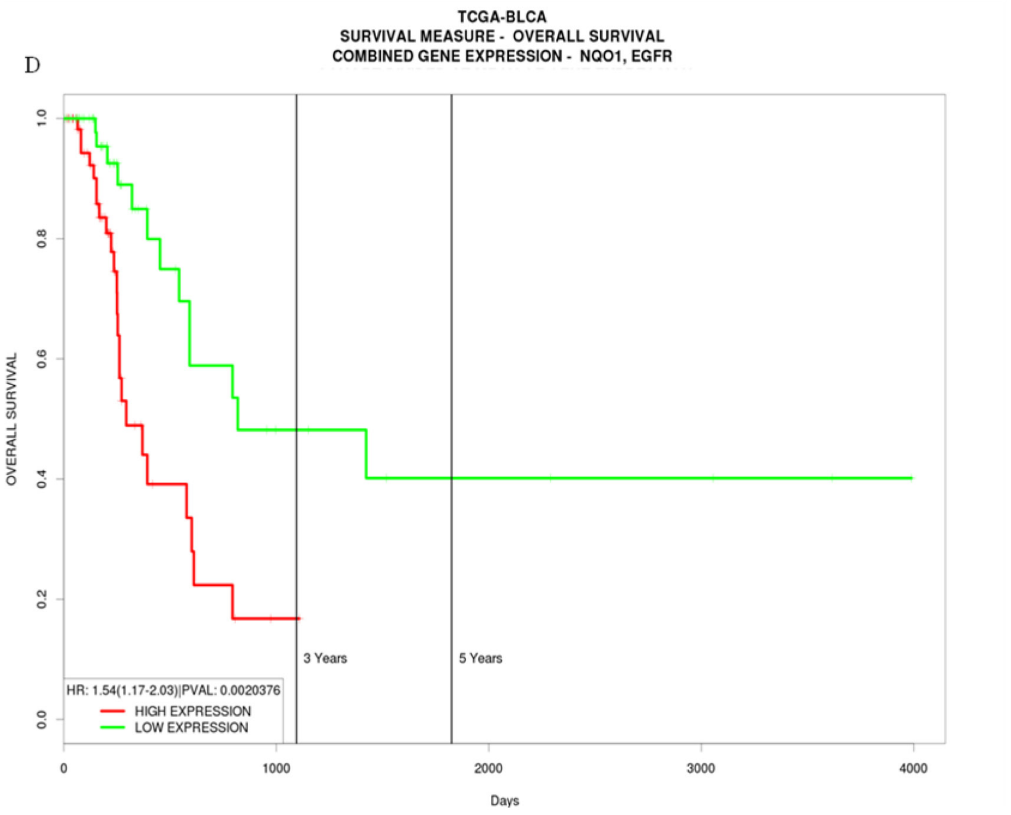

(*Continued*)

E

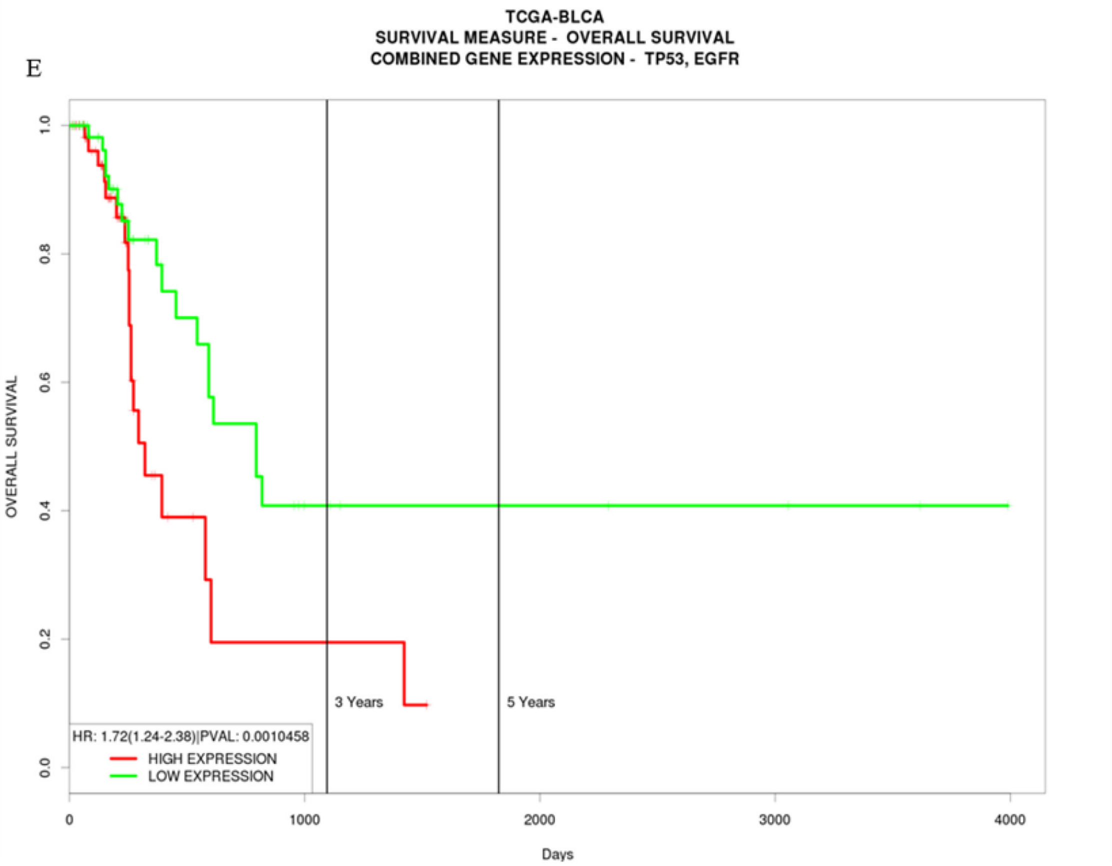

Supplementary Figure 6: Combination effect of HUB genes in "BC and environment" network on overall survival by SynTarget online tool using public TCGA_BLCA dataset (Bladder Urothelial Carcinoma).

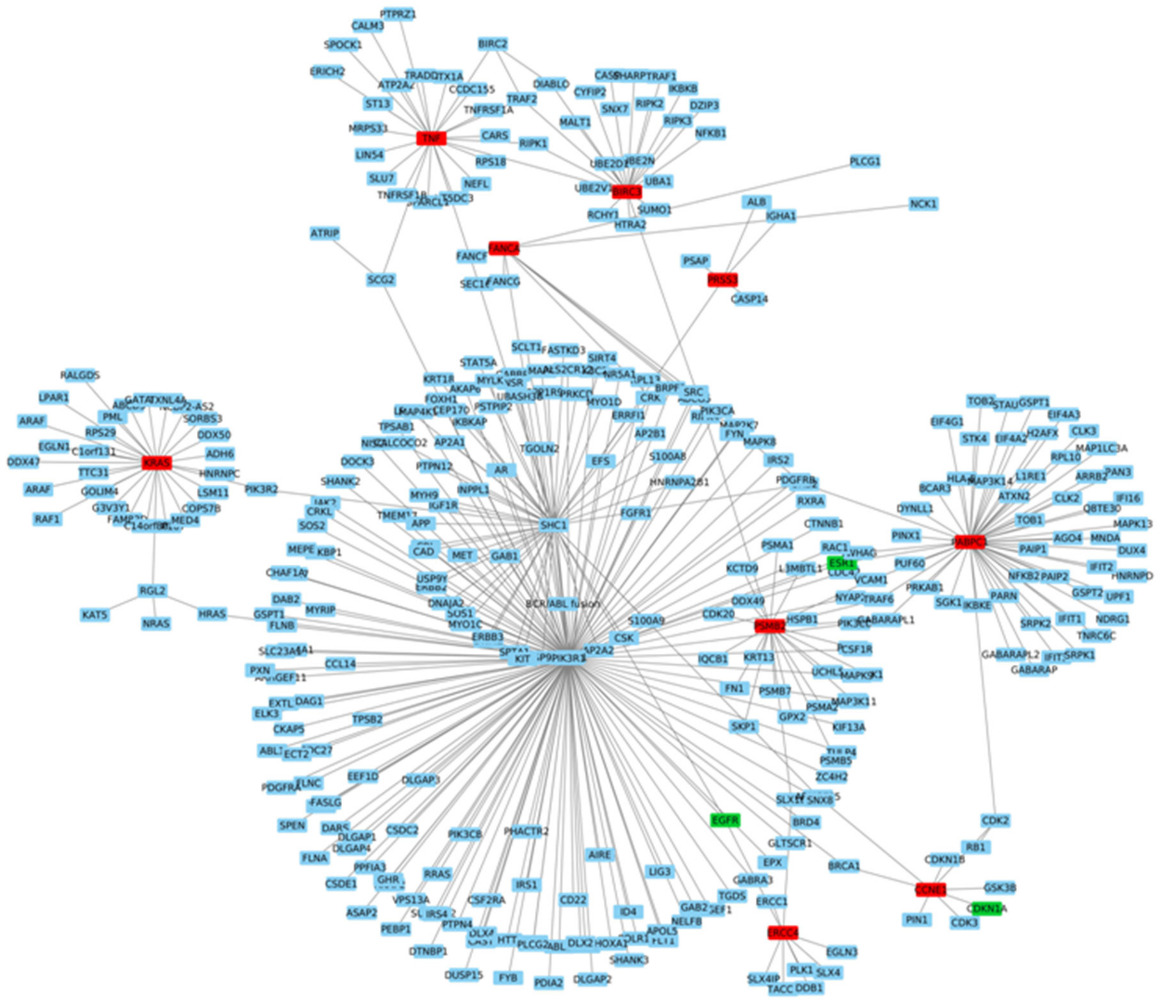

Supplementary Figure 7: Network related to the correlation between proteins modulated from arsenicals in BC. HUB nodes are reported in red and the linked HUB nodes of "BC and environmental" in green.

A

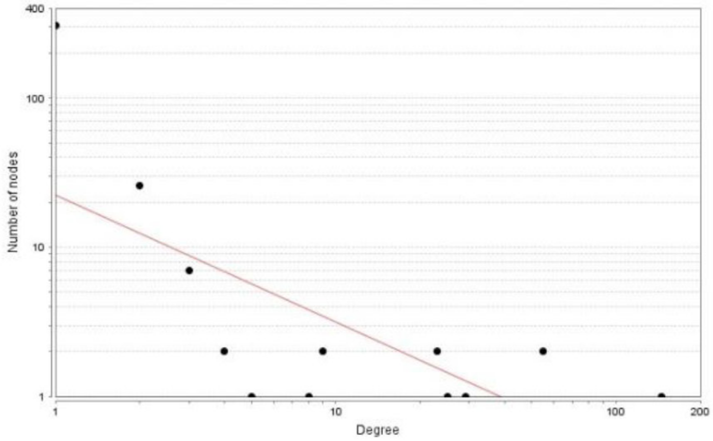

B

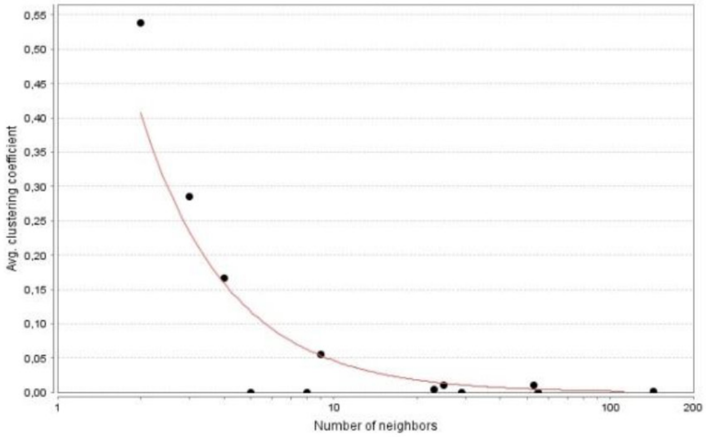

C

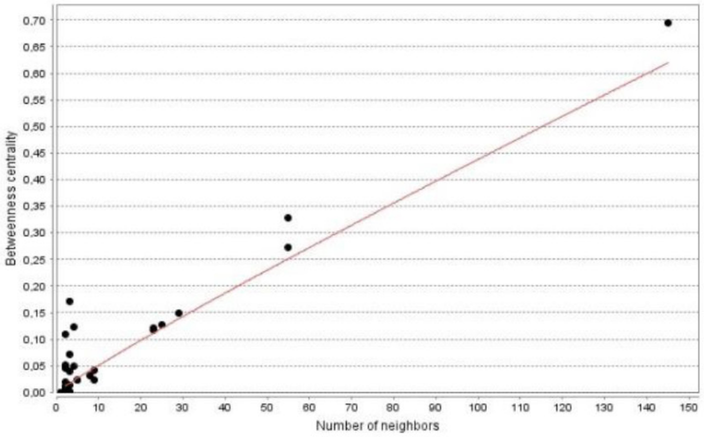

Supplementary Figure 8: Evaluation of topological properties of "BC and arsenicals" network. **(A)** node degree distribution, **(B)** average clustering coefficient, and **(C)** betweenness centrality measure.

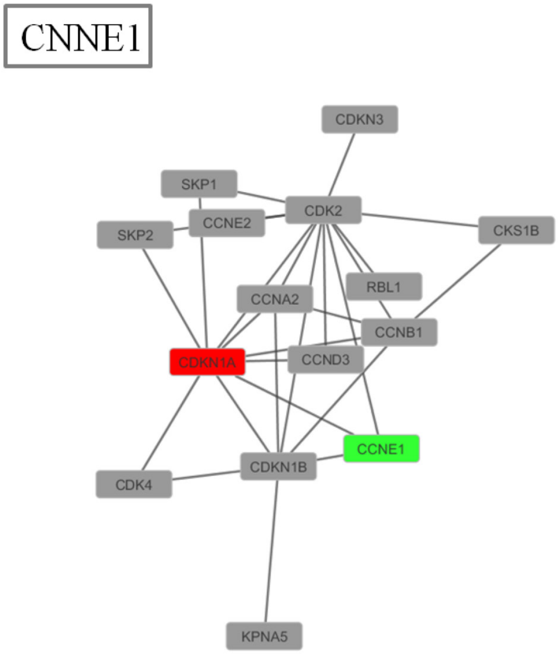

Supplementary Figure 9: Two clusters obtained starting the entire network reported in Supplementary Figure 6.

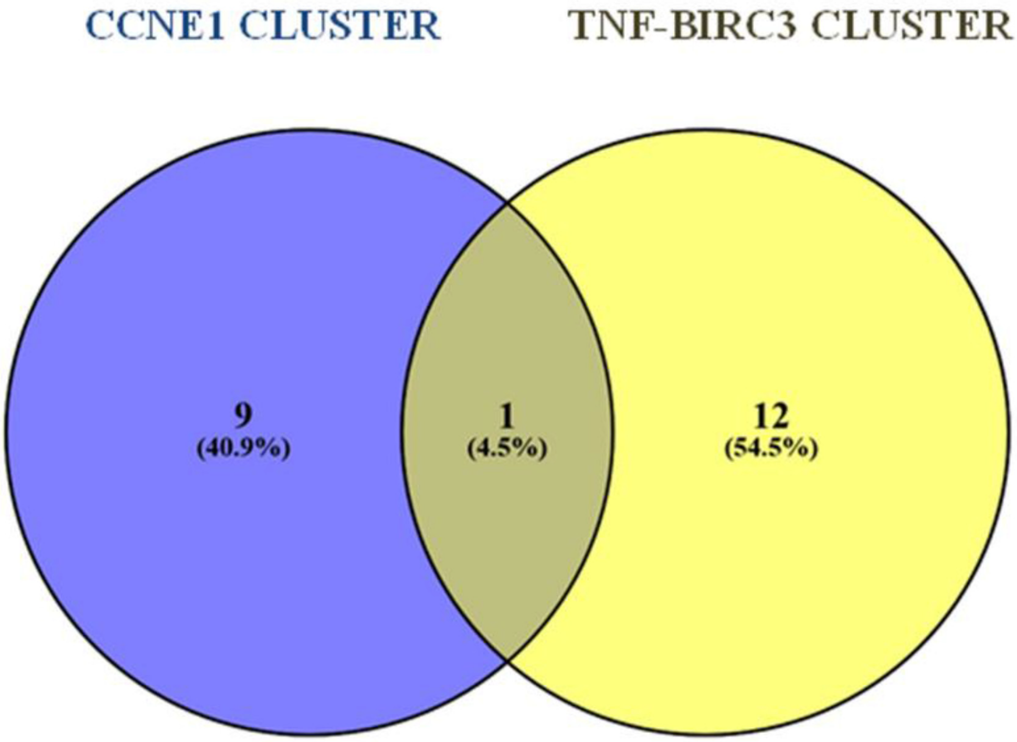

Supplementary Figure 10: Venn diagram evidences the number of common and specific pathways in which the proteins present in two clusters present in Supplementary Figure 8 are involved.

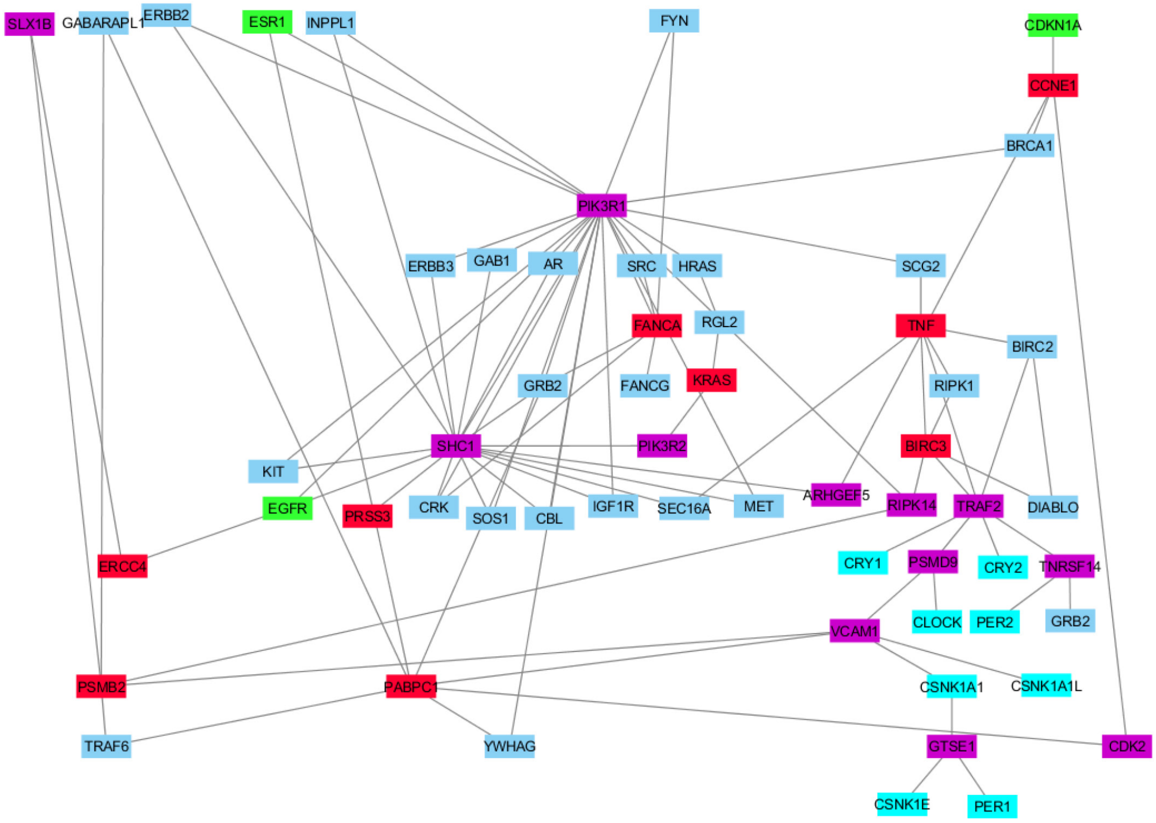

Supplementary Figure 11: Relationship between the HUB-HUB interaction network and circadian nodes. In details, we report HUB nodes in red, circadian nodes in marine green, nodes linking HUB and circadian nodes in purple, hub nodes of bladder environmental in green and the other nodes in cyan.

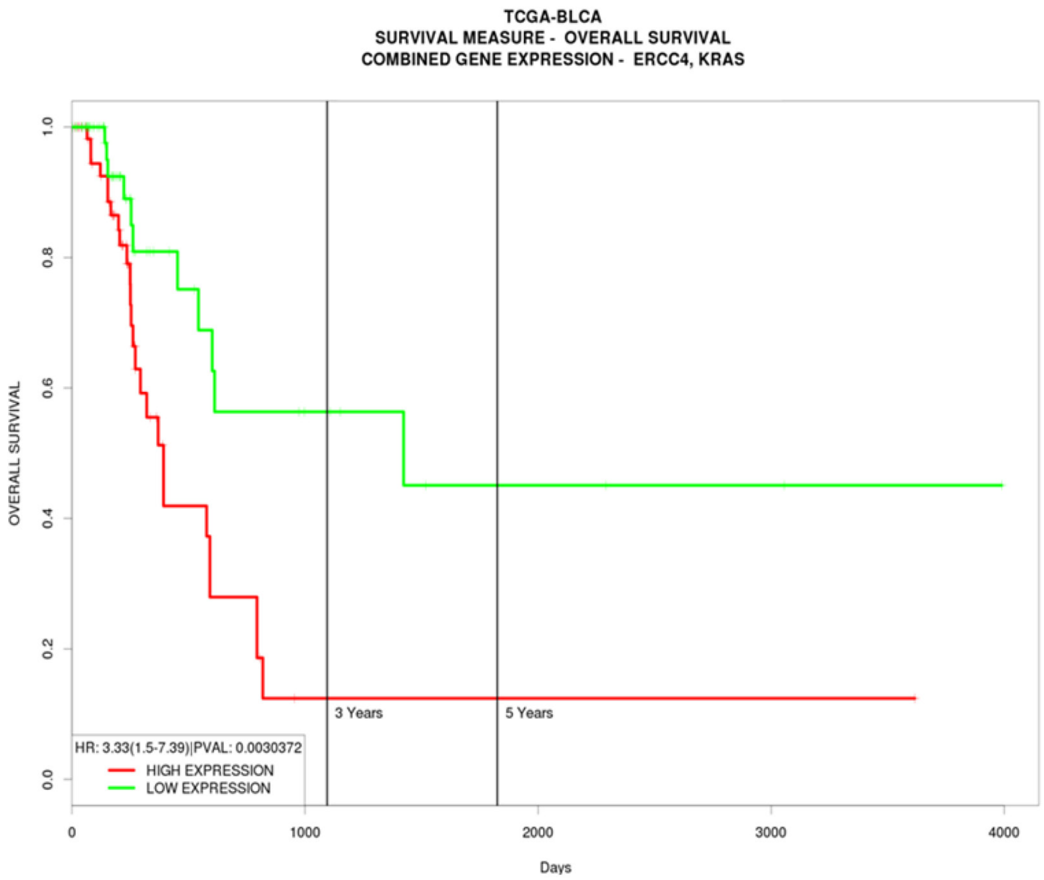

Supplementary Figure 12: Combination effect of HUB genes in "BC and arsenicals" network on overall survival by SynTarget online tool using public TCGA_BLCA dataset (Bladder Urothelial Carcinoma).

Supplementary Table 1: List of the molecules present in the four following categories: arsenicals, smoking, pollutants and others

Categories	Compounds	Chemical properties	Biological functions	Mode of human exposure
**Arsenicals**	arsenite, arsenic, arsenic acid, arsenotriglutathione, sodium arsenite, 4-aminophenylarsenoxide, cacodylic acid, gallium arsenide, monomethylarsonous acid, sodium arsenite	arsenic in the +3 oxidation state	i) inhibition of pyruvate dehydrogenase; ii) inhibition of alpha ketoglutarate dehydrogenase, an enzyme of kreb's cycle; iii) modulation of the cellular metabolism; iv) inhibition of lecithin:cholesterol acyltransferase	water and soil
**Smoking**	3-amino-1-methyl-5H-pyrido(4,3-b)indole, ammonia, benz(a)anthracene, benzo(a) pyrene, 1,2-dimethylhydrazine, tobacco smoke pollution	amine; heterocyclic amines; polycyclic aromatic hydrocarbons;	i) intercalation in DNA; ii) action as DNA methylating agents; iii) inhibitors of the enzymes related to amine metabolism	air
**Pollutants**	carbon dioxide, carbon monoxide, formaldehyde, mercury compounds, nitrogen oxides, particulate matter, sulfur dioxide, vehicle emissions	organic molecules; hydrocarbons; radicals; inorganic molecules;	i) toxicity of heart and central nervous system; ii) decrease of the mitochondrial dehydrogenases activity; iii) alteration of respiratory epithelium innate immunity; iv) stimulation of lactate production	air
**Others**	2-phenylphenol, dieldrin, diethylnitrosamine, diuron, endosulfan, heptachlor epoxide, thiabendazole,	benzene derivates; polycyclic aromatic compound	i) action as endocrine disruptors; ii) alteration of cellcycle phase regulatory proteins; iii) blocking of the reduction of NADP+ to NADPH; iv) inhibition of fumarate reductase	air, soil and water

Moreover we inserted the information related to the chemical properties, the biological functions and the mode of human exposure for the compounds belonging to each category.

Supplementary Table 2: List of proteins modulated by only one sub-group or by two, three or four groups

Categories	Common proteins	Tot. number
All categories	CDH1, KRAS, IGFBP3, GSTM1, IGF1, TP53, IGFBP5, GSTP1, PTGS2, TNF, GPX1, CXCL8, CDKN2A, SOD2, EGFR, NQO1, MYC, CDKN1A, MT2A, BIRC3, FAS, ESR1	22
Arsenicals, pollutants, smoking	PSMB2, GSTZ1	2
Arsenicals, others, smoking	USP7	1
Arsenicals, others, pollutants	ERCC4, FERMT2, ESR2, HDAC4, LIG1, PABPC1, HRAS, EP300, ENO2, RB1, SRC, ERCC2, CCNE1, CREBBP, ATM	15
Others, pollutants, smoking	CSF3, NAT2, RASSF1, ANPEP, CYP4B1, TFRC, NAT1, MPO, MT3, AQP3	10
Arsenicals, pollutants	FGFR3, ANKFY1, MAPK15, RHOA, ISL1, FANCA, ASAP1, IGSF21	8
Arsenicals, others	PRDM2, ATP5D	2
Pollutants, smoking	KMT2C, GSTO2	2
Others, smoking	MTHFR, PSCA	2
Others, pollutants	ESPL1, GGH, TERT, UGT2B7, LAMA2, PRSS3, POLB, POR, KRT16, AS3MT, TRPV1, TRP53, NCOR1, LOXL1, IL2, MT1A	16
Arsenicals	FBXW8	1
Smoking	ANXA2R	1
Pollutants	ASXL2, RALGPS1, STAG2, SMC1B, SMC1A, TYMP, SLC12A7, ARID1A, TACC3, APOBEC3B, MLL2, LOXL4, CALMHM1, NRSN1, EOMES, KMT2A, CHD6, VWA3A, KDM6A	19
Others	BPTF, GLI1, KRT6, HOXA9, IFNA2	5

Supplementary Table 3: Molecular pathways in which the proteins belonging to the four categories are involved

See Supplementary File 1

Supplementary Table 4: List of the common or specific molecular pathways shared by the four categories

See Supplementary File 1

Supplementary Table 5: Molecular pathways in which twenty-two proteins modulated in BC by four chemical sub-groups are involved

Pathways	Proteins involved in each pathway
p53 signaling pathway	FAS, CDKN1A, CDKN2A, IGF1, IGFBP3, TP53
MAPK signaling pathway	FAS, EGFR, TNF, TP53, KRAS, MYC
Apoptosis	FAS, BIRC3, TNF, TP53
ErbB signaling pathway	CDKN1A, EGFR, KRAS, MYC
Cell cycle	CDKN1A, CDKN2A, TP53, MYC
Glutathione metabolism	GSTM1, GSTP1, GPX1

Supplementary Table 6: Summary of the function of the HUB nodes in "BC and environmental" network

See Supplementary File 1

Supplementary Table 7: Common and specific pathways related to four clusters obtained starting from the entire network related to the correlation between "BC and environment"

See Supplementary File 1

Supplementary Table 8: Mutations of HUB nodes involved in the "BC and environment" network

See Supplementary File 1

Supplementary Table 9: List of miRNAs involved in “BC and environment” network

miRNAs involved in “BC and environment” network
hsa-mir-30a-3p
hsa-mir-17-5p
hsa-mir-30c-5p
hsa-mir-125b-5p
hsa-mir-30e-5p
hsa-mir-199a-3p
hsa-mir-7-5p
hsa-mir-644a
hsa-mir-133b
hsa-mir-26a-5p
hsa-mir-101-3p
hsa-mir-520b
hsa-mir-639

Supplementary Table 10: Summary of the functions of the HUB nodes in "BC and arsenical" network

HUB node	Molecular function
**BIRC3 (baculoviral IAP repeat containing 3)**	It inhibits apoptosis by binding to tumor necrosis factor receptor-associated factors by interfering with activation of ICE-like proteases
**CCNE1 (cyclin E1)**	It functions as a regulatory subunit of CDK2, whose activity is required for cell cycle G1/S transition. Itaccumulates at the G1-S phase boundary and is degraded as cells progress through S phase
**ERCC4 (ERCC excision repair 4, endonuclease catalytic subunit)**	It forms the ERCC1-XPF enzyme complex that participates in DNA repair and DNA recombination
**FANCA (Fanconi anemia complementation group A)**	It is a DNA repair protein that may operate in a postreplication repair or a cell cycle checkpoint function. It may be involved in interstrand DNA cross-link repair and in the maintenance of normal chromosome stability
**KRAS (KRAS proto-oncogene)**	It plays an important role in the regulation of cell proliferation, and in promoting oncogenic events by inducing transcriptional silencing of tumor suppressor genes
**PABPC1 (Poly(A) binding protein cytoplasmic 1)**	It shuttles between the nucleus and cytoplasm and its binding to poly(A) promotes ribosome recruitment and translation initiation
**PRSS3 (protease, serine 3)**	It is a trypsinogen, which is active on peptide linkages involving the carboxyl group of lysine or arginine
**PSMB2 (proteasome subunit beta type-2)**	It is one of 28 subunits of proteasome and contributes to form a proteolytic environment for substrate degradation. Its unique structure design prevents random encounter between proteolytic active sites and protein substrate, which makes protein degradation a well-regulated process
**TNF (tumor necrosis factor)**	It is a cytokine that is involved in the regulation of a wide spectrum of biological processes including cell proliferation, differentiation, apoptosis, lipid metabolism, and coagulation

Supplementary Table 11: Common and specific pathways related to two clusters obtained starting from the entire network related to the correlation between "BC and arsenicals"

Pathways	Pathways in which the proteins present in the clusters are involved
**Common cluster CCNE1, TNF-BIRC3**	- Pathways in cancer
**Specific to cluster CCNE1**	- Oocyte meiosis<br>- Epstein-Barr virus infection<br>- FoxO signaling pathway<br>- PI3K-Akt signaling pathway<br>- Measles<br>- Hepatitis B<br>- p53 signaling pathway<br>- Viral carcinogenesis<br>- Cell cycle
**Specific to cluster TNF-BIRC3**	- Apoptosis<br>- NF-kappa B signaling pathway<br>- TNF signaling pathway<br>- Small cell lung cancer<br>- Toxoplasmosis<br>- Adipocytokine signaling pathway<br>- Sphingolipid signaling pathway<br>- Hepatitis C<br>- Tuberculosis<br>- Herpes simplex infection<br>- NOD-like receptor signaling pathway<br>- RIG-I-like receptor signaling pathway

Supplementary Table 12: Mutations of HUB nodes involved in the "BC and arsenicals" network

BC and arsenicals		AA. change
**HUB**	**Mutation type**	
BIRC3	M	R149T
CCNE1	/	/
ERCC4	M	E210K
ERCC4	M	A906P
FANCA	M	L1401V
FANCA	M	S1264Y
FANCA	M	P1222L
FANCA	M	E698K
KRAS	/	/
PABPC1	/	/
PRSS3	/	/
PSMB2	/	/
TNF	/	/

Supplementary Table 13: List of miRNAs involved in "BC and arsenicals" network

miRNAs involved in "BC and arsenicals" network
hsa-mir-17-5p
hsa-mir-520b
hsa-mir-30a-3p
hsa-mir-26a-5p
hsa-mir-7-5p
hsa-mir-125b-5p
hsa-mir-646
