# Supplementary material for: Environment and bladder cancer: molecular analysis by interaction networks
Source: Oncotarget. 2017 May 26;8(39):65240–52. doi: 10.18632/oncotarget.18222 (PMC5630327; doi:10.18632/oncotarget.18222)
Supplement: Supplementary file 2 [file oncotarget-08-65240-s002.doc]

**Supplementary Table 3: Molecular pathways in which the proteins belonging to the four categories are involved**

| **Categories** | **Pathway** | **Genes** |
| --- | --- | --- |
| Arsenicals | Pathways in cancer | CXCL8, CREBBP, EP300, FAS, HRAS, KRAS, RB1, BIRC3, CDH1, CCNE1, CDKN1A, CDKN2A, EGFR, FGFR3, IGF1, PTGS2, RHOA, TP53, MYC |
|  | Hepatitis B | CXCL8, CREBBP, EP300, FAS, HRAS, KRAS, RB1, SRC, CCNE1, CDKN1A, TNF, TP53, MYC |
|  | Viral carcinogenesis | CREBBP, EP300, HRAS, KRAS, RB1, SRC, CCNE1, CDKN1A, CDKN2A, HDAC4, RHOA, TP53, USP7 |
|  | MicroRNAs in cancer | ATM, CREBBP, EP300, HRAS, KRAS, CCNE1, CDKN1A, CDKN2A, EGFR, FGFR3, PTGS2, RHOA, TP53, MYC |
|  | Proteoglycans in cancer | FAS, HRAS, KRAS, SRC, CDKN1A, EGFR, ESR1, IGF1, RHOA, TNF, TP53, MYC |
|  | FoxO signaling pathway | ATM, CREBBP, EP300, HRAS, KRAS, CDKN1A, EGFR, IGF1, SOD2, USP7 |
|  | p53 signaling pathway | ATM, FAS, CCNE1, CDKN1A, CDKN2A, IGF1, IGFBP3, TP53 |
|  | Cell cycle | ATM, CREBBP, EP300, RB1, CCNE1, CDKN1A, CDKN2A, TP53, MYC |
|  | HTLV-I infection | ATM, CREBBP, EP300, HRAS, KRAS, RB1, CDKN1A, CDKN2A, TNF, TP53, MYC |
|  | Thyroid hormone signaling pathway | CREBBP, EP300, HRAS, KRAS, SRC, ESR1, TP53, MYC |
|  | Central carbon metabolism in cancer | HRAS, KRAS, EGFR, FGFR3, TP53, MYC |
|  | Adherens junction | CREBBP, EP300, SRC, CDH1, EGFR, RHOA |
|  | Epstein-Barr virus infection | CREBBP, EP300, RB1, CDKN1A, HDAC4, TP53, USP7, MYC |
|  | ErbB signaling pathway | HRAS, KRS, SRC, CDKN1A, EGFR, MYC |
|  | Rap1 signaling pathway | HRAS, KRAS, SRC, CDH1, EGFR, FGFR3, IGF1, RHOA |
|  | HIF-1 signaling pathway | CREBBP, EP300, CDKN1A, ENO2, EGFR, IGF1 |
|  | Estrogen signaling pathway | HRAS, KRAS, SRC, EGFR, ESR1, ESR2 |
|  | Oxytocin signaling pathway | HRAS, KRAS, SRC, CDKN1A, EGFR, PTGS2, RHOA |
|  | Apoptosis | ATM, FAS, BIRC3, TNF, TP53 |
|  | Transcriptional misregulation in cancer | ATM, CXCL8, CDKN1A, IGF1, IGFBP3, TP53, MYC |
|  | Prolactin signaling pathway | HRAS, KRAS, SRC, ESR1, ESR2 |
|  | MAPK signaling pathway | FAS, HRAS, KRAS, EGFR, FGFR3, TNF, TP53, MYC |
|  | PI3K-Akt signaling pathway | HRAS, KRAS, CCNE1, CDKN1A, EGFR, FGFR3, IGF1, TP53, MYC |
|  | Signaling pathways regulating pluripotency of stem cells | HRAS, ISL1, KRAS, FGFR3, IGF1, MYC |
|  | TGF-beta signaling pathway | CREBBP, EP300, RHOA, TNF, MYC |
|  | NF-kappa B signaling pathway | ATM, CXCL8, BIRC3, PTGS2, TNF |
|  | VEGF signaling pathway | HRAS, KRAS, SRC, PTGS2 |
|  | Sphingolipid signaling pathway | HRAS, KRAS, RHOA, TNF, TP53 |
|  | Long-term potentiation | CREBBP, EP300, HRAS, KRAS |
|  | Focal adhesion | HRAS, SRC, BIRC3, EGFR, IGF1, RHOA |
|  | Regulation of actin cytoskeleton | HRAS, KRAS, SRC, EGFR, FGFR3, RHOA |
|  | Wnt signaling pathway | CREBBP, EP300, RHOA, TP53, MYC |
|  | Ras signaling pathway | HRAS, KRAS, EGFR, FGFR3, IGF1, RHOA |
|  | Gap junction | HRAS, KRAS, SRC, EGFR |
|  | GnRH signaling pathway | HRAS, KRAS, SRC, EGFR |
|  | Endocytosis | ASAP1, HRAS, SRC, EGFR, FGFR3, RHOA |
|  | Melanogenesis | CREBBP, EP300, HRAS, KRAS |
|  | T cell receptor signaling pathway | HRAS, KRAS, RHOA, TNF |
|  | TNF signaling pathway | FAS, BIRC3, PTGS2, TNF |
|  | Chemokine signaling pathway | CXCL8, HRAS, KRAS, SRC, RHOA |
|  | Nucleotide excision repair | LIG1, ERCC2, ERCC4 |
|  | Neurotrophin signaling pathway | HRAS, KRAS, RHOA, TP53 |
|  | Glutathione metabolism | GSTM1, GSTP1, GPX1 |
|  | Natural killer cell mediated cytotoxicity | FAS, KRAS, HRAS, TNF |
|  | NOD-like receptor signaling pathway | CXCL8, BIRC3, TNF |
| Smoking | Pathways in cancer | CXCL8, FAS, KRAS, RASSF1, BIRC3, CDH1, CDKN1A, CDKN2A, EGFR, IGF1, PTGS2, TP53, MYC |
|  | Proteoglycans in cancer | FAS, KRAS, CDKN1A, EGFR, ESR1, IGF1, TNF, TP53, MYC |
|  | p53 signaling pathway | FAS, CDKN1A, CDKN2A, IGF1, IGFBP3, TP53 |
|  | Chemical carcinogenesis | NAT1, NAT2, GSTM1, GSTO2, GSTP1, PTGS2 |
|  | Hepatitis B | CXCL8, FAS, KRAS, CDKN1A, TNF, TP53, MYC |
|  | Glutathione metabolism | ANPEP, GSTM1, GSTO2, GSTP1, GPX1 |
|  | Transcriptional misregulation in cancer | CXCL8, CDKN1A, IGF1, IGFBP3, MPO, TP53, MYC |
|  | MicroRNAs in cancer | KRAS, RASSF1, CDKN1A, CDKN2A, EGFR, PTGS2, TP53, MYC |
|  | FoxO signaling pathway | KRAS, CDKN1A, EGFR, IGF1, SOD2, USP7 |
|  | Apoptosis | FAS, BIRC3, TNF, TP53 |
|  | Central carbon metabolism in cancer | KRAS, EGFR, TP53, MYC |
|  | PI3K-Akt signaling pathway | KRAS, CSF3, CDKN1A, EGFR, IGF1, TP53, MYC |
|  | MAPK signaling pathway | FAS, KRAS, EGFR, TNF, TP53, MYC |
|  | HTLV-I infection | KRAS, CDKN1A, CDKN2A, TNF, TP53, MYC |
|  | Hematopoietic cell lineage | ANPEP, CSF3, TFRC, TNF |
|  | ErbB signaling pathway | KRAS, CDKN1A, EGFR, MYC |
|  | NF-kappa B signaling pathway | CXCL8, BIRC3, PTGS2, TNF |
|  | HIF-1 signaling pathway | CDKN1A, EGFR, IGF1, TFRC |
|  | TNF signaling pathway | FAS, BIRC3, PTGS2, TNF |
|  | Viral carcinogenesis | KRAS, CDKN1A, CDKN2A, TP53, USP7 |
|  | Thyroid hormone signaling pathway | KRAS, ESR1, TP53, MYC |
|  | Cell cycle | CDKN1A, CDKN2A, TP53, MYC |
|  | Caffeine metabolism | NAT1, NAT2 |
|  | Cytokine-cytokine receptor interaction | CXCL8, FAS, CSF3, EGFR, TNF |
|  | NOD-like receptor signaling pathway | CXCL8, BIRC3, TNF |
|  | Oxytocin signaling pathway | KRAS, CDKN1A, EGFR, PTGS2 |
|  | Drug metabolism - cytochrome P450 | GSTM1, GSTO2, GSTP1 |
| Pollutants | Pathways in cancer | CXCL8, CREBBP, EP300, FAS, HRAS, RB1, RASSF1, BIRC3, CDH1, CCNE1, CDKN1A, CDKN2A, EGFR, IGF1, LAMA2, PTGS2, RHOA, TP53, MYC |
|  | Cell cycle | ATM, CREBBP, EP300, RB1, CCNE1, CDKN1A, CDKN2A, ESPL1, STAG2, SMC1A, SMC1B, TP53, MYC |
|  | Hepatitis B | CXCL8, CREBBP, EP300, FAS, HRAS, KRAS, RB1, SRC, CCNE1, CDKN1A, TNF, TP53, MYC |
|  | MicroRNAs in cancer | ATM, CREBBP, EP300, HRAS, RASSF1, CCNE1, CDKN1A, CDKN2A, EGFR, PTGS2, RHOA, TP53, MYC |
|  | Viral carcinogenesis | CREBBP, POLB, EP300, HRAS, KRAS, RB1, SRC, CCNE1, CDKN1A, CDKN2A, HDAC4, RHOA, TP53 |
|  | HTLV-I infection | ATM, CREBBP, POLB, EP300, HRAS, KRAS, PB1, CDKN1A, CDKN2A, IL2, TERT, TNF, TP53, MYC |
|  | p53 signaling pathway | ATM, FAS, CCNE1, CDKN1A, CDKN2A, IGF1, IGFBP3, TP53 |
|  | Proteoglycans in cancer | FAS, HRAS, KRAS, SRC, CDKN1A, EGFR, ESR1, IGF1, RHOA, TNF, TP53, MYC |
|  | Transcriptional misregulation in cancer | ATM, CXCL8, CDKN1A, IGF1, IGFBP3, KDM6A, KMT2A, MPO, NCOR1, TP53, MYC |
|  | Thyroid hormone signaling pathway | CREBBP, EP300, HRAS, KRAS, SRC, ESR1, NCOR1, TP53, MYC |
|  | FoxO signaling pathway | ATM, CREBBP, EP300, HRAS, KRAS, CDKN1A, EGFR, IGF1, SOD2 |
|  | Chemical carcinogenesis | NAT1, NAT2, UGT2B7, GSTM1, GSTO2, GSTP1, PTGS2 |
|  | HIF-1 signaling pathway | CREBBP, EP300, CDKN1A, ENO2, EGFR, IGF1, TFRC |
|  | Central carbon metabolism in cancer | HRAS, KRAS, EGFR, FGFR3, TP53, MYC |
|  | PI3K-Akt signaling pathway | HRAS, KRAS, CSF3, CCNE1, CDKN1A, EGFR, IGF1, IL2, LAMA2, TP53, MYC |
|  | Adherens junction | CREBBP, EP300, SRC, CDH1, EGFR, RHOA |
|  | Glutathione metabolism | ANPEP, GSTM1, GSTO2, GSTP1, GPX1 |
|  | ErbB signaling pathway | HRAS, KRAS, SRC, CDKN1A, EGFR, MYC |
|  | Estrogen signaling pathway | HRAS, KRAS, SRC, EGFR, ESR1, ESR2 |
|  | Apoptosis | ATM, FAS, BIRC3, TNF, TP53 |
|  | Oxytocin signaling pathway | HRAS, KRAS, SRC, CDKN1A, EGFR, PTGS2, RHOA |
|  | Rap1 signaling pathway | HRAS, KRAS, SRC, CDH1, EGFR, FGFR3, IGF1, RHOA |
|  | Prolactin signaling pathway | HRAS, KRAS, SRC, ESR1, ESR2 |
|  | TGF-beta signaling pathway | CREBBP, EP300, RHOA, TNF, MYC |
|  | Drug metabolism - other enzymes | NAT1, NAT2, UGT2B7, TYMP |
|  | NF-kappa B signaling pathway | ATM, CXCL8, BIRC3, PTGS2, TNF |
|  | Signaling pathways regulating pluripotency of stem cells | HRAS, KRAS, ISL1, FGFR3, IGF1, MYC |
|  | MAPK signaling pathway | FAS, HRAS, KRAS, EGFR, FGFR3, TNF, TP53, MYC |
|  | Focal adhesion | HRAS, SRC, BIRC3, EGFR, IGF1, LAMA2, RHOA |
|  | T cell receptor signaling pathway | HRAS, KRAS, IL2, RHOA, TNF |
|  | VEGF signaling pathway | HRAS, KRAS, SRC, PTGS2 |
|  | Oocyte meiosis | CCNE1, ESPL1, IGF1, SMC1A, SMC1B |
|  | Ras signaling pathway | HRAS, KRAS, RASSF1, EGFR, FGFR3, IGF1, RHOA |
|  | Long-term potentiation | CREBBP, EP300, HRAS, KRAS |
|  | Drug metabolism - cytochrome P450 | UGT2B7, GSTM1, GSTO2, GSTP1 |
|  | Sphingolipid signaling pathway | HRAS, KRAS, RHOA, TNF, TP53 |
|  | Metabolism of xenobiotics by cytochrome P450 | UGT2B7, GSTM1, GSTO2, GSTP1 |
|  | Graft-versus-host disease | FAS, IL2, TNF |
|  | Endocytosis | ASAP1, HRAS, SRC, EGFR, RHOA, TFRC |
| Others | Hepatitis B | CXCL8, CREBBP, EP300, FAS, HRAS, KRAS, RB1, SRC, CCNE1, CDKN1A, IFNA2, TNF, TP53, MYC |
|  | Pathways in cancer | CXCL8, CREBBP, EP300, FAS, GLI1, HRAS, KRAS, RB1, RASSF1, BIRC3, CDH1, CCNE1, CDKN1A, CDKN2A, EGFR, IGF1, LAMA2, PTGS2, TP53, MYC |
|  | Viral carcinogenesis | CREBBP, POLB, EP300, HRAS, KRAS, RB1, SRC, CCNE1, CDKN1A, CDKN2A, HDAC4, TP53, USP7 |
|  | HTLV-I infection | ATM, CREBBP, POLB, EP300, HRAS, KRAS, RB1, CDKN1A, CDKN2A, IL2, TERT, TNF, TP53, MYC |
|  | Cell cycle | ATM, CREBBP, EP300, RB1, CCNE1, CDKN1A, CDKN2A, ESPL1, TP53, MYC |
|  | p53 signaling pathway | ATM, FAS, CCNE1, CDKN1A, CDKN2A, IGF1, IGFBP3, TP53 |
|  | FoxO signaling pathway | ATM, CREBBP, EP300, HRAS, KRAS, CDKN1A, EGFR, IGF1, SOD2, USP7 |
|  | MicroRNAs in cancer | ATM, CREBBP, EP300, HRAS, KRAS, RASSF1, CCNE1, CDKN1A, CDKN2A, EGFR, PTGS2, TP53, MYC |
|  | Thyroid hormone signaling pathway | CREBBP, EP300, HRAS, KRAS, SRC, ESR1, NCOR1, TP53, MYC |
|  | Proteoglycans in cancer | FAS, HRAS ,KRAS, SRC, CDKN1A, EGFR, ESR1, IGF1, TNF, TP53, MYC |
|  | Transcriptional misregulation in cancer | ATM, CXCL8, CDKN1A, IGF1, IGFBP3, MPO, NCOR1, TP53, MYC |
|  | PI3K-Akt signaling pathway | HRAS, KRAS, CSF3, CCNE1, CDKN1A, EGFR, IGF1, IFNA2, IL2, LAMA2, TP53, MYC |
|  | HIF-1 signaling pathway | CREBBP, EP300, CDKN1A, ENO2, EGFR, IGF1, TFRC |
|  | Chemical carcinogenesis | NAT1, NAT2, UGT2B7, GSTM1, GSTP1, PTGS2 |
|  | ErbB signaling pathway | HRAS, KRAS, SRC, CDKN1A, EGFR, MYC |
|  | Epstein-Barr virus infection | CREBBP, EP300, RB1, CDKN1A, HDAC4, TP53, USP7, MYC |
|  | Estrogen signaling pathway | HRAS, KRAS, SRC, EGFR, ESR1, ESR2 |
|  | Apoptosis | ATM, FAS, BIRC3, TNF, TP53 |
|  | Central carbon metabolism in cancer | HRAS, KRAS, EGFR, TP53, MYC |
|  | Prolactin signaling pathway | HRAS, KRAS, SRC, ESR1, ESR2 |
|  | Adherens junction | CREBBP, EP300, SRC, CDH1, EGFR |
|  | NF-kappa B signaling pathway | ATM, CXCL8, BIRC3, PTGS2, TNF |
|  | Jak-STAT signaling pathway | CREBBP, EP300, CSF3, IFNA2, IL2, MYC |
|  | Glutathione metabolism | ANPEP, GSTM1, GSTP1, GPX1 |
|  | Oxytocin signaling pathway | HRAS, KRAS, SRC, CDKN1A, EGFR, PTGS2 |
|  | Cytokine-cytokine receptor interaction | CXCL8, FAS, CSF3, EGFR, IFNA2, IL2, TNF |
|  | VEGF signaling pathway | HRAS, KRAS, SRC, PTGS2 |
|  | Long-term potentiation | CREBBP, EP300, HRAS, KRAS |
|  | Natural killer cell mediated cytotoxicity | FAS, HRAS, KRAS, IFNA2, TNF |
|  | MAPK signaling pathway | FAS, HRAS, KRAS, EGFR, TNF, TP53, MYC |
|  | Focal adhesion | HRAS, SRC, BIRC3, EGFR, IGF1, LAMA2 |
|  | Rap1 signaling pathway | HRAS, KRAS, SRC, EGFR, CDH1, IGF1 |
|  | Graft-versus-host disease | FAS, IL2, TNF |
|  | TGF-beta signaling pathway | CREBBP, EP300, TNF, MYC |
|  | Hematopoietic cell lineage | ANPEP, CSF3, TFRC, TNF |
|  | Gap junction | HRAS, KRAS, SRC, EGFR |
|  | Allograft rejection | FAS, IL2, TNF |
|  | GnRH signaling pathway | HRAS, KRAS, SRC, EGFR |
|  | Caffeine metabolism | NAT1, NAT2 |

**Supplementary Table 4: List of the common or specific molecular pathways shared by the four categories**

| **Categories** | **Common pathways** |
| --- | --- |
| Arsenicals, smoking, pollutant, others | Pathways in cancer  Hepatitis B  Viral carcinogenesis  MicroRNAs in cancer  Proteoglycans in cancer  FoxO signaling pathway  p53 signaling pathway  Cell cycle  HTLV-I infection  Thyroid hormone signaling pathway  Central carbon metabolism in cancer  ErbB signaling pathway  HIF-1 signaling pathway  Oxytocin signaling pathway  Apoptosis  Transcriptional misregulation in cancer  MAPK signaling pathway  PI3K-Akt signaling pathway  NF-kappa B signaling pathway  Glutathione metabolism |
| Arsenicals, smoking | TNF signaling pathway  NOD-like receptor signaling pathway |
| Arsenicals only | Regulation of actin cytoskeleton  Wnt signaling pathway  Melanogenesis  Chemokine signaling pathway  Nucleotide excision repair  Neurotrophin signaling pathway |
| Arsenicals, pollutant | Signaling pathways regulating pluripotency of stem cells  Sphingolipid signaling pathway  Ras signaling pathway  Endocytosis  T cell receptor signaling pathway |
| Arsenicals, pollutant, others | Adherens junction  Rap1 signaling pathway  Estrogen signaling pathway  Prolactin signaling pathway  TGF-beta signaling pathway  VEGF signaling pathway  Long-term potentiation  Focal adhesion |
| Arsenicals, others | Epstein-Barr virus infection  Gap junction  GnRH signaling pathway  Natural killer cell mediated cytotoxicity |
| Smoking, others | Hematopoietic cell lineage  Caffeine metabolism  Cytokine-cytokine receptor interaction |
| Smoking, pollutants, others | Chemical carcinogenesis |
| Smoking, pollutant | Drug metabolism - cytochrome P450 |
| Pollutant only | Drug metabolism - other enzymes  Oocyte meiosis  Metabolism of xenobiotics by cytochrome P450 |
| Pollutant, others | Graft-versus-host disease |
| Others only | ak-STAT signaling pathway  Allograft rejection |

**Supplementary Table 6: Summary of the function of the HUB nodes in “BC and environmental” network**

| **HUB node** | **Molecular function** |
| --- | --- |
| **CDKN1A (cyclin dependent kinase inhibitor 1A)** | It is a potent cyclin-dependent kinase inhibitor that binds to and inhibits the activity of cyclin-cyclin-dependent kinase2. It functions as a regulator of cell cycle progression at G1, and, when it interacts with proliferating cell nuclear antigen, a DNA polymerase accessory factor, it plays a regulatory role in S phase DNA replication and DNA damage repair. |
| **CDKN2A (cyclin-dependent kinase inhibitor 2A)** | It is capable of inducing cell cycle arrest in G1 and G2 phases and of acting as a tumor suppressor. It binds to MDM2 and blocks its nucleocytoplasmic shuttling by sequestering it in the nucleolus. |
| **CXCL8 (C-X-C motif chemokine ligand 8)** | It is a major mediator of the inflammatory response and a potent angiogenic factor |
| **EGFR (epidermal growth factor receptor)** | It is a cell surface protein that binds to epidermal growth factor and induces receptor dimerization and tyrosine autophosphorylation by leading to cell proliferation. |
| **ESR1 (estrogen receptor 1)** | It is a ligand-activated transcription factor composed of hormone binding, DNA binding, and activation domain of transcription involved in sexual development and reproductive function. |
| **FAS (Fas cell surface death receptor)** | It plays a central role in the physiological regulation of programmed cell death, and has been implicated in the pathogenesis of various malignancies and diseases of the immune system |
| **GSTM1 (glutathione S-transferase Mu 1)** | It functions in the detoxification of electrophilic compounds, including carcinogens, therapeutic drugs, environmental toxins and products of oxidative stress, by conjugation with glutathione |
| **GSTP1 (glutathione S-transferase Pi 1)** | It plays an important role in detoxification by catalyzing the conjugation of many hydrophobic and electrophilic compounds with reduced glutathione. |
| **IGFBP3 (insulin like growth factor binding protein 3)** | It circulates in the plasma, prolonging the half-life of IGFs and altering their interaction with cell surface rece |
| **KRAS (KRAS proto-oncogene)** | It plays an important role in the regulation of cell proliferation, and in promoting oncogenic events by inducing transcriptional silencing of tumor suppressor genes |
| **MYC (v-myc avian myelocytomatosis viral oncogene )** | It plays a role in cell cycle progression, apoptosis and cellular transformation. It functions as a transcription factor that regulates transcription of specific target genes |
| **NQO1 (NAD(P)H quinone dehydrogenase 1)** | It reduces quinones to hydroquinones. Its enzymatic activity prevents the one electron reduction of quinones that results in the production of radical species. |
| **SOD2 (superoxide dismutase 2)** | It is a mitochondrial protein that binds to the superoxide byproducts of oxidative phosphorylation and converts them to hydrogen peroxide and diatomic oxygen |
| **TNF (tumor necrosis factor)** | It is a cytokine that is involved in the regulation of a wide spectrum of biological processes including cell proliferation, differentiation, apoptosis, lipid metabolism, and coagulation |
| **TP53 (tumor protein P53)** | It is a tumor suppressor protein containing transcriptional activation, DNA binding, and oligomerization domains. It responds to diverse cellular stresses to regulate expression of target genes, thereby inducing cell cycle arrest, apoptosis, senescence, DNA repair, or changes in metabolism |

**Supplementary Table 7: Common and specific pathways related to four clusters obtained starting from the entire network related to the correlation between “BC and environment”**

| **Pathways** | **Pathways in which the proteins present in the clusters are involved** |
| --- | --- |
| **Common cluster EGFR, MYC** | - Cell cycle  - Epstein-Barr virus infection |
| **Common cluster ESR1, MYC** | - RNA transport |
| **Common cluster EGFR, ESR1** | - Proteoglycans in cancer |
| **Specific to cluster ESR1** | -mRNA surveillance pathway  -Thyroid hormone signaling pathway  -Ribosome biogenesis in eukaryotes  -Spliceosome  -Ribosome |
| **Specific to cluster EGFR** | - Regulation of actin cytoskeleton  - Pathogenic Escherichia coli infection  - Endocrine and other factor-regulated - calcium reabsorption  - Calcium signaling pathway  - Choline metabolism in cancer  - Protein processing in endoplasmic reticulum  - Gap junction  - Fc gamma R-mediated phagocytosis  - Ras signaling pathway  - Hepatitis B  - Insulin signaling pathway  - Prolactin signaling pathway  - Neurotrophin signaling pathway  - Chemokine signaling pathway  - Rap1 signaling pathway  - Viral carcinogenesis  - PI3K-Akt signaling pathway  - MAPK signaling pathway  - Adherens junction  - Estrogen signaling pathway  - HIF-1 signaling pathway  - MicroRNAs in cancer  - Focal adhesion  - Endocytosis  - Bacterial invasion of epithelial cells  - ErbB signaling pathway |
| **Specific to Cluster MYC** | - Lysine degradation  - Oxidative phosphorylation  - Nucleotide excision repair  - Biosynthesis of antibiotics  - Citrate cycle (TCA cycle)  - Propanoate metabolism  - Carbon metabolism  - DNA replication  - Proteasome  - Mismatch repair |
| **Specific to Cluster GSTP1-NQO1** | - TNF signaling pathway  - FoxO signaling pathway  - Regulation of autophagy  - RIG-I-like receptor signaling pathway |

**Supplementary Table 8: Mutations of HUB nodes involved in the “BC and environment” network**

|  | **BC and environment** | |  |  |
| --- | --- | --- | --- | --- |
| **HUB** |  | **Mutation type** | **AA. change** | |
| CDKN1A |  | M | G132C |  |
| CDKN1A |  | M | D136H |  |
| CDKN1A |  | M | G61V |  |
| CDKN2A |  | M | H83Y |  |
| CDKN2A |  | M | D108N |  |
| CDKN2A |  | M | E119K |  |
| CDKN2A |  | M | L31V |  |
| CXCL8 |  | M | Q35E |  |
| EGFR |  | M | R836H |  |
| EGFR |  | M | T384S |  |
| EGFR |  | M | E1079K |  |
| ESR1 |  | / | / |  |
| FAS |  | / | / |  |
| GSTM1 |  | / | / |  |
| GSTP1 |  | M | G146V |  |
| GSTP1 |  | M | R138P |  |
| IGFBP3 |  | / | / |  |
| KRAS |  | / | / |  |
| MYC |  | M | F22L |  |
| NQO1 |  | M | P187S |  |
| SOD2 |  | / | / |  |
| TNF |  | / | / |  |
| TP53 |  | M | R248Q |  |
| TP53 |  | M | R273S |  |
| TP53 |  | M | H193Y |  |
| TP53 |  | M | R248P |  |
| TP53 |  | M | C176F |  |
| TP53 |  | M | C242F |  |
| TP53 |  | M | C238F |  |
| TP53 |  | M | K132N |  |
| TP53 |  | M | Y220C |  |
| TP53 |  | M | S241F |  |
| TP53 |  | M | R282W |  |
| TP53 |  | M | G245V |  |
| TP53 |  | M | L194F |  |
| TP53 |  | M | R248W |  |
| TP53 |  | M | P151H |  |
| TP53 |  | M | R280T |  |
| TP53 |  | M | G245S |  |
| TP53 |  | M | R280K |  |
| TP53 |  | M | R175H |  |
| TP53 |  | M | H214R |  |
| TP53 |  | M | R273C |  |
| TP53 |  | M | A159P |  |
| TP53 |  | M | E171K |  |
| TP53 |  | M | L257Q |  |
| TP53 |  | M | E285V |  |
| TP53 |  | M | A159V |  |
| TP53 |  | M | D148N |  |
| TP53 |  | M | G279R |  |
| TP53 |  | M | E285K |  |
| TP53 |  | M | E271K |  |
| TP53 |  | M | R158H |  |
| TP53 |  | M | P152L |  |
| TP53 |  | M | C277F |  |
| TP53 |  | M | K139N |  |
| TP53 |  | M | W146S |  |
| TP53 |  | M | A161T |  |
| TP53 |  | M | E204Q |  |
| TP53 |  | M | P72R |  |
